# Carbon Dots Crosslinked Egg White Hydrogel for Tissue Engineering

**DOI:** 10.1002/advs.202404702

**Published:** 2024-09-20

**Authors:** Jun Wu, Josh Haipeng Lei, Moxin Li, Aiping Zhang, Yuan Li, Xiao Liang, Senio Campos de Souza, Zhen Yuan, Chunming Wang, Guokai Chen, Tzu‐Ming Liu, Chu‐Xia Deng, Zikang Tang, Songnan Qu

**Affiliations:** ^1^ Joint Key Laboratory of the Ministry of Education Institute of Applied Physics and Materials Engineering University of Macau Macau 999078 P. R. China; ^2^ Department of Physics and Chemistry Faculty of Science and Technology University of Macau Macao 999078 P. R. China; ^3^ MOE Frontier Science Centre for Precision Oncology University of Macau Macao 999078 P. R. China; ^4^ Faculty of Health Sciences University of Macau Macau 999078 P. R. China; ^5^ Institute of Chinese Medical Sciences & State Key Laboratory of Quality Research in Chinese Medicine University of Macau Macau 999078 P. R. China

**Keywords:** carbon dots, egg white, hair follicle regeneration, protein hydrogels, wound healing

## Abstract

Egg white (EW)‐derived hydrogels hold promise as biomaterials for in vitro cell culture due to their ability to mimic the extracellular matrix. However, their highly cross‐linked structures restrict their potential for in vivo applications, as they are unable to integrate dynamically with tissues before degradation. In this study, this limitation is addressed by introducing carbon dots (CDs) as cross‐linking agents for EW in a dilute aqueous solution. The resulting CDs‐crosslinked EW hydrogel (CEWH) exhibits tensile strength comparable to that of skin tissue and features a large pore structure that promotes cell infiltration. Subcutaneous implantation of CEWH demonstrated excellent integration with surrounding tissue and a degradation rate aligned with the hair follicles (HFs) regeneration cycle. This allows the long‐term regeneration and establishment of an M2 macrophage‐dominated immune microenvironment, which in turn promotes the re‐entry of HFs into the anagen phase from the telogen phase. Additionally, CEWH demonstrated potential as a wound dressing material. Overall, this study paves the way for utilizing EW as a versatile biomaterial for tissue engineering.

## Graphical abstract

A CDs‐crosslinked egg white hydrogel (CEWH) is synthesized from chicken egg white (EW) and carbon dots (CDs) for modulating long‐term immune‐mediated tissue engineering. The introduction of CDs as cross‐linkers promotes the unfolding and connection of EW protein peptide chains in dilute solutions, thereby effectively avoiding self‐crosslinking and self‐aggregation of EW.

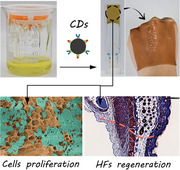

## 1 Introduction

In situ tissue engineering leverages the inherent regenerative potential of the organism for tissue repair.^[^ ^1^, ^2^ ^]^ The design of biomaterial‐derived scaffolds for in situ tissue engineering requires not only the modulation of the extracellular immune microenvironment to facilitate regeneration but also necessitates the balancing of the relationship between material degradation and structure with tissue integration.^[^ ^3^ ^]^ These properties collectively ensure the provision of a stable physical foundation for the long‐term infiltration of immune cells within the scaffolds, thereby facilitating successful tissue repair. Biocompatible scaffolds derived from natural materials that meet the aforementioned criteria are a promising area of research.

Egg white (EW), abundant in essential nutrients, is a readily available and crucial nutritional resource for human health.^[^ ^4^, ^5^ ^]^ A myriad of biologically active compounds (ovalbumin, ovomucoid, lysozyme, derived hydrolyzed peptides, etc.) contained in EW are more favorable to be absorbed by cells, and in parallel exhibit effective immune regulation capacity, thus making EW as a promising natural material for biomedical applications.^[^ ^6^, ^7^ ^]^ Hydrogel scaffolds derived from EW through self‐crosslinking have been demonstrated to provide a matrix for cellular growth and proliferation.^[^ ^8^ ^]^ However, the existing synthetic approaches for EW‐based scaffolds often result in highly cross‐linked protein chains,^[^ ^9^ ^]^ leading to reduced biological activity and suboptimal tensile strength, which ultimately renders them unsuitable for use in regenerative medicine within a living organism. It is therefore imperative to develop a new method to fabricate EW‐based hydrogels with low‐density, enhanced tensile strength, degradation rates compatible with tissue integration, and preserved immune regulation functions.

Carbon dots (CDs) are renowned for their exceptional biocompatibility, low toxicity, and adaptable optical characteristics.^[^ ^10^, ^11^, ^12^ ^]^ Their ultra‐small size (less than 10 nm) and abundant surface functional groups make them promising candidates as linking agents for bonding with biomolecules to create novel supramolecular architectures.^[^ ^11^ ^]^ Recent studies have demonstrated that the incorporation of CDs into synthetic polymer networks, such as poly(vinyl alcohol) or poly(methylacrylic acid), can facilitate the formation of hydrogels with improved mechanical properties.^[^ ^12^, ^13^, ^14^ ^]^ However, unbalanced degradation rates or potential safety concerns regarding these synthetic polymers in vivo remain major hurdles for clinical applications.

This study describes a facile heating approach to fabricate EW‐based hydrogels (CEWH) utilizing CDs as crosslinkers. Unlike directly heated EW, which results in an opaque, densely crosslinked hydrogel (referred to as EW hydrogel), and diluted EW aqueous solutions that fail to form hydrogels upon heating, the incorporation of CDs enables the preparation of transparent, homogeneous, and loosely porous CEWH with good tensile strength. During the thermal gelation process, the diluted EW protein chains can fully unfold and interact with CDs through hydrogen or covalent bonds. This interaction results in the formation of a low‐density yet stable nano‐linked porous structure, which effectively prevents extensive self‐crosslinking of EW proteins while preserving their inherent bioactive components.

Neither pure EW hydrogel nor CEWH elicited a strong immune response in vivo. However, the smaller pore structure and accelerated degradation of pure EW hydrogel resulted in a loss of tissue integration and impaired cell infiltration growth prior to degradation. In contrast, the CEWH, crosslinked with CDs, exhibited a larger pore structure and a prolonged degradation period. These characteristics facilitated the sustained inward infiltration and accumulation of cells and vasculature before material degradation. The in vivo prolonged degradation rate of the CEWH was found to be well‐matched with the hair follicles (HFs) regeneration cycle, making it a suitable biomaterial for HFs regenerative medicine. Following subcutaneous implantation of CEWH, macrophages were recruited and continuously proliferated in the M2 phenotype via the interleukin‐6/signal transducer and activator of transcription 3 (IL‐6/STAT3) pathway within the implant scaffolds over several weeks.^[^ ^15^ ^]^ This continuously modulated the subcutaneous immune microenvironment, thereby promoting long‐lasting HFs regeneration. These findings indicate the potential of EW as a regenerative material and suggest a subcutaneous implantation approach for the induction of HFs growth in adult mice. Moreover, the capacity of CEWH to stimulate the aggregation of M2 macrophages and the regeneration of HFs was shown to facilitate wound healing and the restoration of skin function, positioning it as a promising biomaterial for broader tissue repair and regeneration applications.

## 2 Results and Discussion

**Figure 1:**
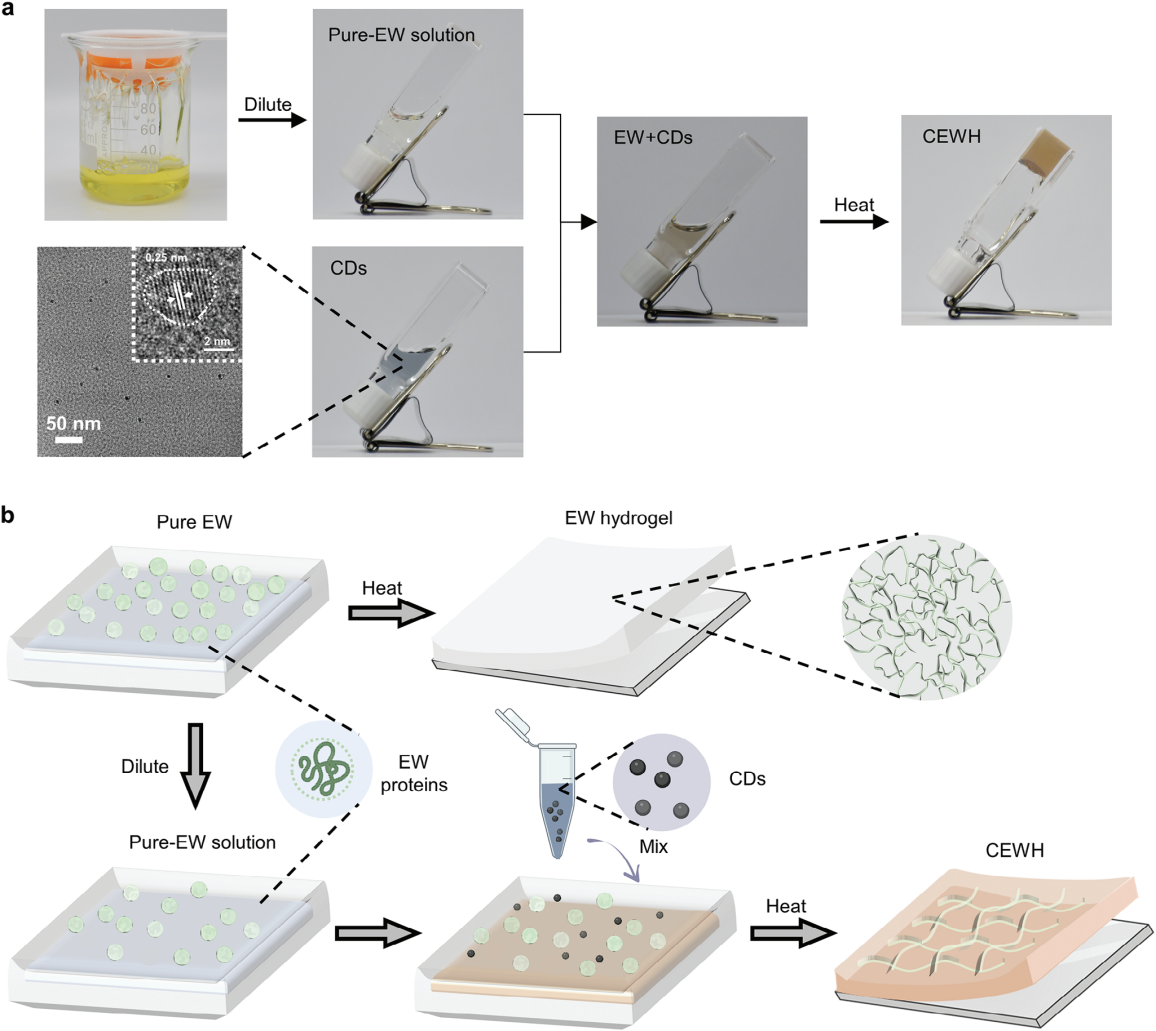
Schematic illustration of CEWH formation process. a) The actual synthetic steps of CEWH. The CDs were combined with a diluted pure‐EW solution to create an EW + CD mixture, which was subsequently heated to produce a transparent hydrogel‐CEWH. b) Depiction of the possible molecular‐level interactions during the formation of EW‐based hydrogels with or without CDs addition.

As previously described, CDs were synthesized, resulting in nanoparticles rich in surface functional groups such as carboxyl and amino groups.^[^ ^16^ ^]^ As shown in **Figure** **1**a, a transparent, non‐flowing hydrogel (CEWH) was formed by mixing diluted egg white (EW) with a CDs aqueous solution under high‐temperature heating. Notably, under the same reaction conditions, the pure dilute EW solution remained in a liquid state and did not form a hydrogel (Figure S1, Supporting Information). This highlights the crucial role of CDs in the formation process of CEWH.

**Figure 2:**
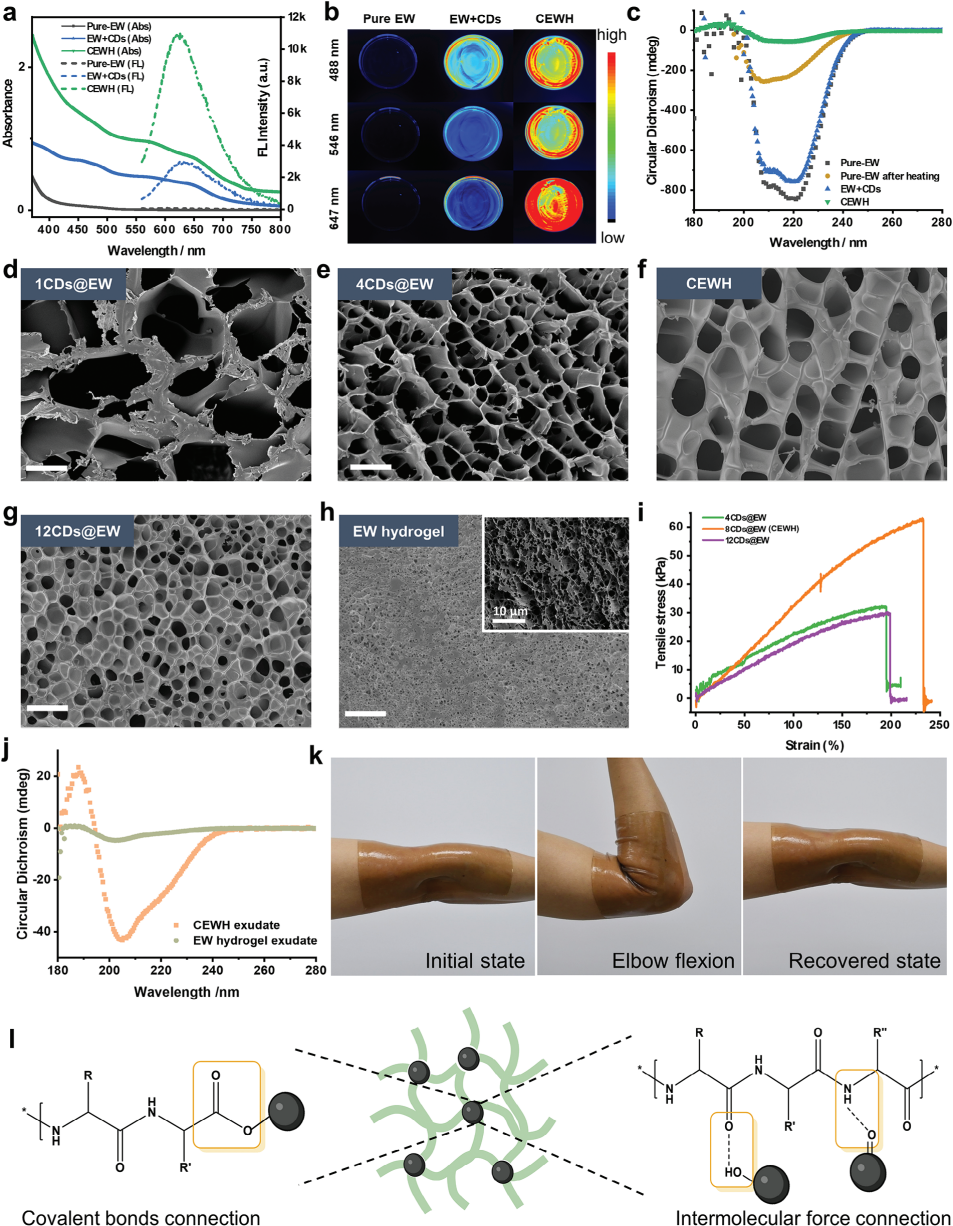
The mechanistic investigation of CDs cross‐linked EW proteins. a) Absorption and fluorescence spectra (excitation at 589 nm) of a pure‐EW solution, EW + CDs solution, and CEWH. b) Fluorescence images of a pure‐EW solution, EW + CDs solution, and CEWH (excited at 488, 546, and 647 nm). c) Circular dichroism spectra of a pure‐EW solution before and after heating, EW + CDs solution, and CEWH. d–g) SEM images of xerogels: 1CDs@EW, 4CDs@EW, CEWH (8CDs@EW), and 12CDs@EW. Scale bars: 20 µm. h) SEM images of EW hydrogel. Scale bar: 20 µm. The inset in (h) provided a magnified view of the pore structure of the EW hydrogel. i) Tensile stress–strain curves for 4CDs@EW, 8CDs@EW (CEWH), and 12CDs@EW. The initial dimensions of the specimen were 16.0 mm × 4.0 mm × 1.0 mm. Tensile tests were conducted at a constant rate of 20 mm min^−1^ at room temperature. j) Circular dichroism spectra of exudates from CEWH and EW hydrogel, obtained by immersing 7 g of each in 6 mL of water for 48 h. k) Photographs of large‐scale CEWH (20 cm × 15 cm) applied to a human elbow joint undergoing elbow flexion. l) A proposed mechanism for the formation of a stretchable and transparent network structure using CDs and EW peptide chains.

The optical properties of three samples were measured: a dilute aqueous solution of pure EW (a pure‐EW solution), a dilute aqueous solution containing both EW and CDs (EW + CDs solution), and CEWH. In comparison to the pure‐EW solution, the EW + CDs solution displayed a broad absorption band in the visible light range, which originated from the CDs, suggesting effective dispersion of the CDs within the EW proteins (**Figure** **2**a). Upon heating and subsequent gelation, the absorption bands of CEWH exhibited a notable increase in intensity, accompanied by an extended tail into the near‐infrared region. This phenomenon could be attributed to the formation of supramolecular structures within the hydrogel, leading to enhanced light scattering. Moreover, when exposed to light at 488 nm, 546 nm, and 647 nm, the fluorescence intensity from CEWH was markedly higher than that from the EW + CDs solution (Figure 2b; Figure S2a‐c, Supporting Information). We hypothesized that thermally unfolded EW protein chains, which bonded to the CDs’ surface during gelation, hindered contact between CDs and water molecules, preventing energy dissipation caused by water molecules and leading to the observed enhancement in fluorescence.

To validate this hypothesis, circular dichroism spectra were measured in order to monitor structural changes in EW proteins with and without the presence of CDs during heating (Figure 2c). In the absence of CDs, the “pure‐EW solution” was heated to obtain “pure‐EW after heating”; in the presence of CDs, “EW + CDs” were heated to obtain “CEWH”. As anticipated, the CDs themselves did not display any evidence of circular dichroism. Prior to heating, the pure‐EW solution exhibited a strong circular dichroism signal, attributable to its high concentration of biomolecules. It is noteworthy that the addition of CDs resulted in a slight reduction in the intensity of the circular dichroism signal observed in the EW + CDs solution, when compared to the pure‐EW solution, suggesting weak interactions between CDs and EW protein molecules that may promote unfolding. Following heat treatment, the intensity of the circular dichroism signal from the pure‐EW solution decreased by approximately two‐thirds. As no flocculation was observed, this reduction was likely attributable to the denaturation of EW protein molecules induced by heat. In contrast, CEWH exhibited a significantly greater loss of circular dichroism signals, indicating that CDs facilitated the complete unfolding of EW protein chains during heating, leading to the formation of an irreversible nano‐linked hydrogel structure.

Fourier transform infrared spectroscopy (FT‐IR) was performed to investigate the interactions between CDs and EW protein chains (Figure S2d, Supporting information). The broad absorption bands observed at 3250–3700 cm^−1^ correspond to stretching vibrations of N─H and O─H groups.^[^ ^17^ ^]^ The sharp peaks observed at 1653 and 1538 cm^−1^ were attributed to amide I band (C═O) and amide II band (C─N stretch coupled with N─H bending) vibrations, respectively.^[^ ^18^, ^19^ ^]^ Compared to the EW + CDs sample, the CEWH xerogel exhibited a slight reduction in the stretching vibration signals of N─H and O─H, suggesting the occurrence of dehydration reactions between surface groups on CDs (carboxy, hydroxyl, amino, etc.) and exposed functional groups (carboxy, amino, etc.) on the peptide chains. The reduced C═O and C─N vibration signals may be due to amide bond breakage resulting from the intensified hydrolysis of EW proteins following thermal treatment. The FT‐IR results were further supported by XPS results. The high‐resolution O 1s XPS spectrum of both EW + CDs and CEWH xerogel confirmed the presence of C─O/C─OH (532.2 eV) and O─C═O (531.1 eV) bonds (Figure S2e,f, Supporting Information).^[^ ^20^, ^21^ ^]^ Compared to EW + CDs, CEWH xerogel showed an increased O─C═O bond and a decreased C─O/C─OH bond, indicating that partial hydroxyl groups were dehydrated to ester groups during the thermal gelation.

Given that CDs were demonstrated to act as crosslinkers in the formation of hydrogel, the amount of CDs added would significantly influence the hydrogel formation process. Aqueous CDs of varying concentrations (1, 4, 8, and 12 mg ml^−1^) were added to the pure‐EW solution, resulting in the formation of hydrogels designated as 1CDs@EW, 4CDs@EW, 8CDs@EW (referred to as CEWH), and 12CDs@EW, respectively. Scanning electron microscopy (SEM) revealed that all hydrogels exhibited a porous structure, with significant differences in pore size distribution (Figure 2d–g). 1CDs@EW exhibited the largest pore size, with an average diameter of ≈45 µm. As the amounts of CDs increased, the prepared hydrogels exhibited a more homogeneous continuous porous network, accompanied by a gradual decrease in pore sizes. The pore diameters for 4CDs@EW, 8CDs@EW, and 12CDs@EW were ≈18, 13, and 7 µm, respectively. According to the morphological differences, we investigated the swelling behavior of these hydrogels. It was observed that following a period of 17 h of swelling, the volume of hydrogels (4CDs@EW, 8CDs@EW, and 12CDs@EW) increased greatly, while the structure of the 1CDs@EW was found to be compromised due to its inherently unstable nature (Figure S3, Supporting Information). Furthermore, after 68 h of swelling, the maximum swelling ratios of 4CDs@EW, 8CDs@EW, and 12CDs@EW were ≈430%, 270%, and 170%, respectively (Figure S4d, Supporting Information). The pore size distribution characteristics of these four hydrogels indicate that the addition of more CDs results in a reduction in pore diameter and a corresponding decrease in the maximum equilibrium swelling ratio. We speculate that the differences in pore diameter and swelling ratio of these hydrogels are attributable to varying degrees of interconnection among protein peptide chains.

The rheological and mechanical behavior of the four hydrogels was also investigated. The storage modulus (G′) was found to be significantly higher than the corresponding loss modulus (G″) when measured through amplitude sweep experiments at a constant frequency of 5 Hz, thereby confirming the elastic properties of the four hydrogels (Figure S4a, Supporting Information). As the shear stress increased, both moduli reached a specific stress threshold (yield stress), at which point they suddenly intersected and exhibited a sharp decline. The yield stress increased with the addition of CDs, with 8CDs@EW exhibiting the highest yield stress of 1.36 kPa (Figure S4b, Supporting Information). This validates our hypothesis that CDs, acting as cross‐linking agents, can enhance the mechanical stability of hydrogels, and that adjusting the amounts of CDs can tune the mechanical properties of the gels. In dynamic frequency sweep experiments (1–35 Hz), the G′ values of 4CDs@EW, 8CDs@EW, and 12CDs@EW were approximately one order of magnitude higher than G″, indicating stable gel states (Figure S4c, Supporting Information). However, the G′ value of 1CDs@EW fell below G″ at ≈32 Hz, indicating an unstable hydrogel structure. In terms of tensile performance measurement, 1CDs@EW was too fragile to undergo tensile tests, thus we only evaluated the tensile properties of 4CDs@EW, 8CDs@EW, and 12CDs@EW. Among these, 8CDs@EW exhibited the best tensile performance, with an ultimate tensile strength of ≈62 kPa and an elongation of 230%, significantly exceeding that of 4CDs@EW and 12CDs@EW (Figure 2i). Based on the results above, we inferred that CDs, acting as cross‐linking agents, could influence the interactions and cross‐linking degree of protein peptide chains. The rational concentration of CDs was found to be a crucial factor in achieving the desired tensile strength of the CDs‐linked EW hydrogel. An insufficient amount of CDs is unable to facilitate effective interconnectivity between adjacent protein peptide chains, resulting in an unstable and loose network gel structure. Considering the mechanical properties of synthesized hydrogel, 8CDs@EW (CEWH) was chose for further investigation.

In light of the findings, a mechanism for hydrogel formation was proposed (Figure 2l). During heating of the EW + CDs solution, the EW proteins unfolded, exposing functional groups, such as amino, carbonyl and carboxyl groups. These groups then bonded with surface functional groups on the CDs via non‐covalent or covalent bonds, including intermolecular hydrogen bonding or dehydration reactions. The CDs served as crosslinking agents, facilitating the formation of a robust 3D and porous network structure by connecting the unfolded protein chains.

To compare bioapplication value, a white, opaque EW hydrogel was produced by directly heating raw chicken EW (Figure 1b). SEM revealed that the pure EW hydrogel exhibited a markedly more condensed porous network with a pore size of ≈1 µm (Figure 2h). Without CDs, the process is known as denaturation or unfolding, which is fundamentally distinct from CDs‐mediated chemical linking. The higher EW content in the EW hydrogel resulted in highly cross‐linked protein chains. Exudate solutions were collected by immersing 7 g of CEWH or EW hydrogel in 6 mL of water for 48 h. Circular dichroism spectra revealed that the CEWH exudate exhibited a significantly stronger dichroism signal than the EW hydrogel exudate (Figure 2j), indicating a higher capacity for the release of bioactive molecules from CEWH. Given the observed morphological differences, we inferred that the highly cross‐linked protein density within the EW hydrogel restricted the movement of the contained bioactive molecules, while the loose and larger porous structure conferred by CDs in CEWH allowed greater freedom for release, which is particularly advantageous for medical materials.^[^ ^22^ ^]^

Besides, the high elasticity and tensile properties allowed a large CEWH patch (20 cm × 15 cm) to comfortably adhere to human skin and withstand the tensile forces exerted by routine movements (Figure 2k; Video S2, Supporting Information). Notably, the formed free surface of CEWH exhibited a sealed structure, while both the stripped surface and cross‐section displayed a porous mesh‐like arrangement (Figure S5, Supporting Information). The stripped surface also exhibited a higher water contact angle in comparison to the freely formed surface, which can be attributed to its higher roughness. This distinctive structure potentially enhanced moisture retention within CEWH. Additionally, due to its hydrophilic protein composition, CEWH effectively absorbed water droplets and demonstrated robust skin adhesion (Figure S6 and Video S3, Supporting Information). Taken together, the skin‐conforming tensile properties and distinctive structural composition of CEWH highlight its potential as a promising biomaterial for artificial skin dressings.

**Figure 3:**
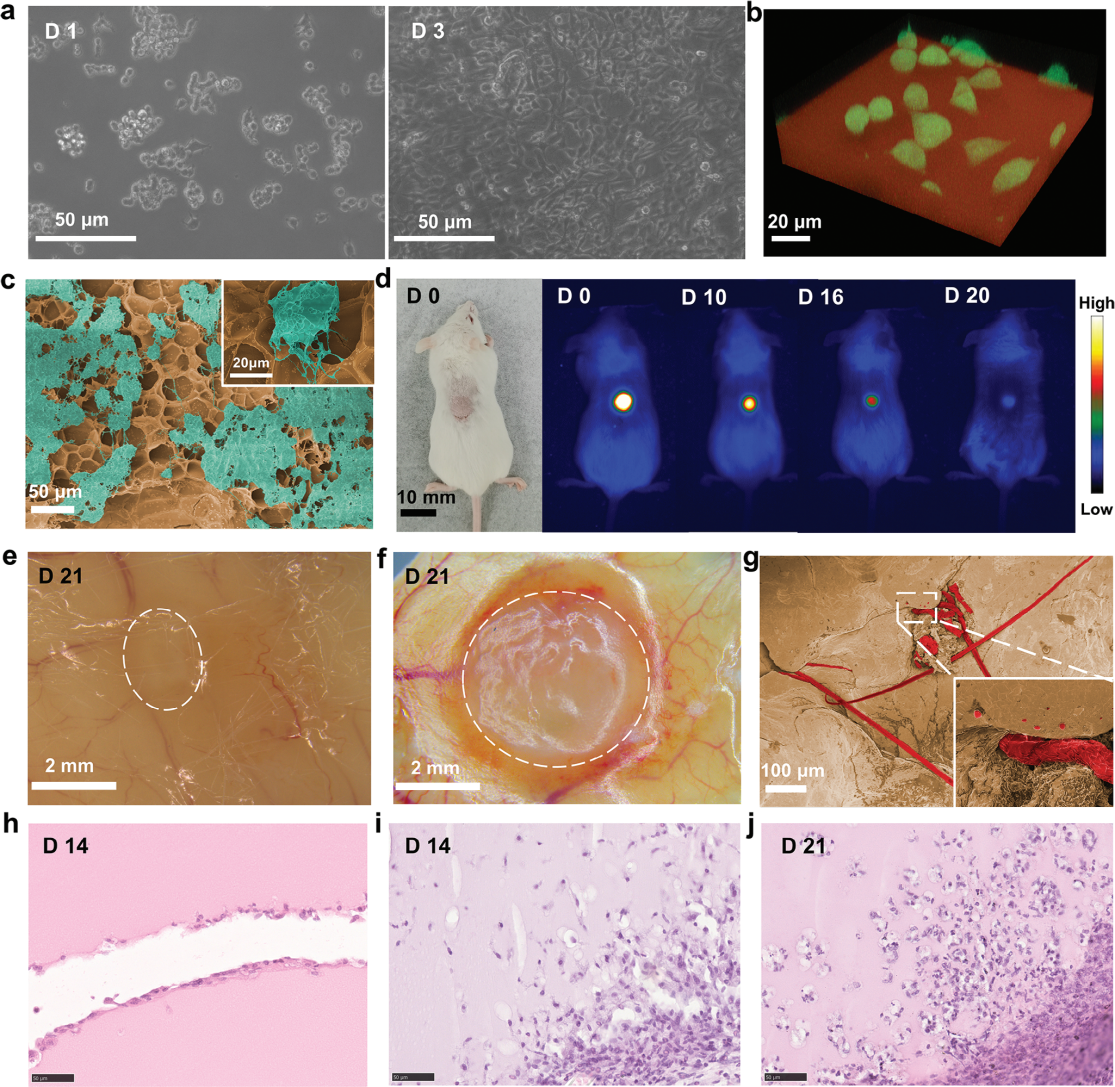
Characterization of CEWH biocompatibility and its interaction with cells. a) Optical images of MDA‐MB‐231 cells incubated on the stripped surface of CEWH, captured on days 1 and 3. b) 3D fluorescence image (TPF) of Hela‐GFP cells (green) cultured on CEWH (red), with fluorescence generated by a 960 nm femtosecond laser. c) SEM image of CEWH xerogel after a 3‐day incubation with MDA‐MB‐231 cells. d) In vivo fluorescence images of a mouse with a subcutaneous CEWH implant, excited with a 589 nm laser. Images were collected using a 610 nm long‐pass optical filter at various time points post‐implantation. Excised mouse skin with e) an EW hydrogel implant and f) a CEWH implant, respectively, captured using a stereomicroscope (Leica MZ10 F) on day 21 post‐implantation. All implants were 5 mm × 5 mm × 2 mm. g) SEM images of the CEWH implant xerogel after 21 days in vivo post‐implantation. The yellow region corresponds to the CEWH implant, and the red regions represent red blood cells and blood vessels. Inset shows a high‐definition magnified SEM image. h–j) Hematoxylin and eosin staining of the subcutaneous (h) EW hydrogel implant on day 14 post‐implantation and (i, j) CEWH implant on days 14 and 21 post‐implantation (scale bars: 50 µm for all histological images). The pinkish‐purple regions represent the stained hydrogel implants.

The cytotoxicity and cell growth‐promoting capabilities of both CEWH exudate and CEWH were evaluated in vitro using three cell lines: mouse fibroblasts (L929), mouse embryonic fibroblasts (NIH3T3) and human breast cancer cells (MDA‐MB‐231), which provides a comprehensive evaluation of CEWH's impact on cell viability and proliferation. The CEWH exudate solution was prepared by immersing a 4 cm × 4 cm × 2 mm CEWH sample in 5 mL PBS for 72 h. L929 and NIH3T3 cells cultured with various concentrations of CEWH exudate solution exhibited high survival rates (>80%), indicating minimal cytotoxicity (Figure S7a, Supporting Information). Additionally, excellent cell attachment and proliferation were observed on the CEWH surface. The density of MDA‐MB‐231 cells cultured on CEWH increased significantly within three days (**Figure** **3**a; Figure S7b, Supporting Information). This observation was corroborated by 3D two‐photon fluorescence (TPF) imaging, which revealed vigorous cellular growth on CEWH (Figure 3b). The SEM image of lyophilized CEWH seeded with MDA‐MB‐231 cells showed that cells with numerous tentacles attached to the porous mesh‐like structure (Figure 3c). Given that EW is rich in proteins and essential nutrients, we conclude that CEWH serves as an excellent scaffold for facilitating cell growth and proliferation.

Having established CEWH's capacity to promote cell growth and proliferation in vitro, we proceeded to investigate its compatibility and degradability in vivo. A subcutaneous implantation mouse model was employed, wherein a 5 mm × 5 mm × 2 mm CEWH patch was implanted on the backs of BALB/c mice. To prevent interference from surgical incisions during the implantation process, the hydrogels were positioned in the skin area away from the incision site, with only the implantation area clipped during sampling collection.

The fluorescent property of CDs enables CEWH to emit red light (peaking at ≈640 nm) when excited by 589 nm laser irradiation. This enabled us to monitor the in vivo degradation rate of the CEWH based on the red fluorescence intensity at the implant site (Figure 3d). The red fluorescent signal persisted for 1 week, gradually diminished over 2 weeks, and became undetectable by the 3 week. These results suggest that the accelerated degradation of CEWH starts around day 16 and persists for at least 20 days.

Mouse skin with implants was excised and examined under a stereomicroscope on day 21. Typically, foreign body implantation induces a dense collagen fibrous capsule around the implant, hindering metabolic exchange, cell signaling, and healing.^[^ ^23^, ^24^ ^]^ However, CEWH integrated well into the surrounding skin tissue, exhibiting no excessive inflammation, pus formation, or difficulty in removal (Figure 3f; Figure S8a,b, Supporting Information). The implantation region showed fine and twisted neovascularization proliferating around a tissue fiber membrane encapsulating the CEWH, as further confirmed by in vivo photoacoustic imaging (Figure S8c, Supporting Information). SEM analysis of the excised CEWH implant on day 21 revealed capillaries attached to and even penetrating into the implant (Figure 3g).

For comparison, a subcutaneously implanted EW hydrogel of the same size was examined under a stereomicroscope to assess its degradation rate and biocompatibility. In contrast to CEWH, the EW hydrogel exhibited a faster degradation rate, becoming nearly undetectable by the naked eye on day 21 (Figure 3e). Remarkably, the fibrous membrane and blood vessels surrounding the gel showed no significant differences compared to the adjacent normal tissue. These results indicated excellent histocompatibility for both the EW hydrogel and CEWH.

Hematoxylin and eosin (H&E) staining was performed on the implantation site. On day 14, inflammatory cells were observed to adhere to the outer region of the residual EW hydrogel without penetrating the interior region (Figure 3h). This is likely due to its higher protein density and smaller pore size structure caused by highly cross‐linked protein chains. In contrast, extensive infiltration of inflammatory cells into the CEWH implants was observed on day 14, with these cells continuing to proliferate within the implant on day 21 (Figure 3i,j). By functioning as a scaffold, CEWH provided spatial space for sustained inflammatory cell accumulation and growth, continuously modulating the immune microenvironment of the implanted area. More importantly, new blood vessels were observed to penetrate the CEWH, which could provide the necessary nutrients for the growth of interior cells.^[^ ^25^ ^]^ Taken together, our results demonstrated that the CDs‐linked EW strategy did not increase the immunogenicity of EW. Instead, it significantly prolonged the in vivo degradation rate of the EW‐derived biomaterial and enabled CEWH to exhibit comprehensive cellular and vascular infiltration, thereby facilitating the integration with the surrounding tissues.

**Figure 4:**
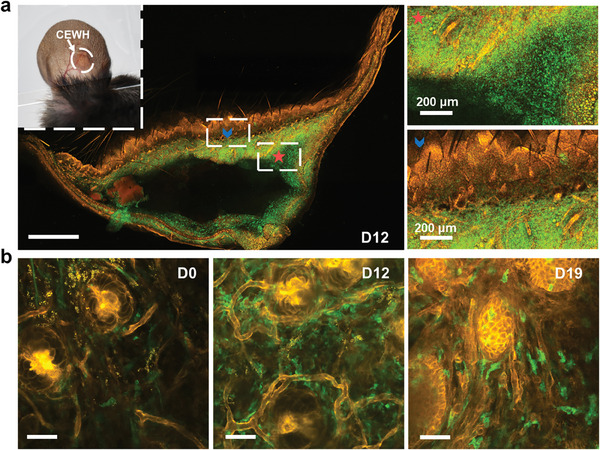
Deep tissue imaging of 3 mm × 3 mm × 1 mm CEWH implants in LysM‐Cre‐mT/mG mouse ears using TPF microscopy. a) TPF images (scale bar: 1 mm) of a tissue section from the mouse ear containing a subcutaneous CEWH implant. The section was collected on day 12 post‐implantation. Two femtosecond lasers with distinct wavelengths were used for excitation: 960 nm for the green channel capturing macrophages and collagen fibers signals, and 1100 nm for the red channel capturing blood vessel signals. The inset shows a photograph of the experimentally operated mouse ear. b) TPF images (scale bars: 50 µm) of the HFs at the implant site, captured at different time points post‐CEWH implantation.

To visualize the subcutaneous immune microenvironment regulated by CEWH, we established an ear implantation model on LysM‐Cre‐mT/mG mice, where macrophages specifically exhibited green fluorescence upon irradiation with a 960‐nm femtosecond laser.^[^ ^26^ ^]^ The thinness of the mouse ear skin allows for in vivo two‐photon fluorescence confocal imaging. Compared to the normal skin area, by day 12 and day 19 post‐implantation, numerous green fluorescent signals were observed around the CEWH implants, indicating predominant macrophage infiltration within CEWH (**Figure** **4**a; Figure S9, Supporting Information). The accumulation of macrophages around and within the CEWH altered the immune microenvironment of the implant site. We also observed an increased density of subcutaneous capillaries in the implant sites (Figure 4b; Figure S10, Supporting Information), consistent with the in vivo photoacoustic imaging results. Additionally, compared to normal skin area, the number and size of HFs within the implant sites substantially increased over time (Figure S11, Supporting Information).

The anagen phase of the mouse HFs growth cycle lasts ≈2 weeks.^[^ ^27^ ^]^ Given the immune microenvironment regulation ability and observed degradation rate of CEWH in vivo (at least 14 days), our findings suggested that the EW‐derived hydrogels could circumvent the need for material degradation and integration that matches the HFs’ regeneration cycle. To validate this hypothesis, we again used a subcutaneous implantation Balb/c mouse model. Skin samples were collected from the implantation area at different time points across three groups: untreated control, EW hydrogel‐implantation group, and CEWH‐implantation group. Histological staining with H&E and Masson's trichrome revealed distinct patterns of HFs regeneration for each group. Compared to the untreated control group (Figure S12a, Supporting Information), the EW hydrogel group exhibited a transient increase in regenerated HFs within the adipose layer on day 14. However, these HFs completely gradually vanished by days 21 and further days 28, coinciding with the degradation of EW hydrogel and presenting only in a minimally thickened dermal layer (Figure S12b‐d, Supporting Information).

**Figure 5:**
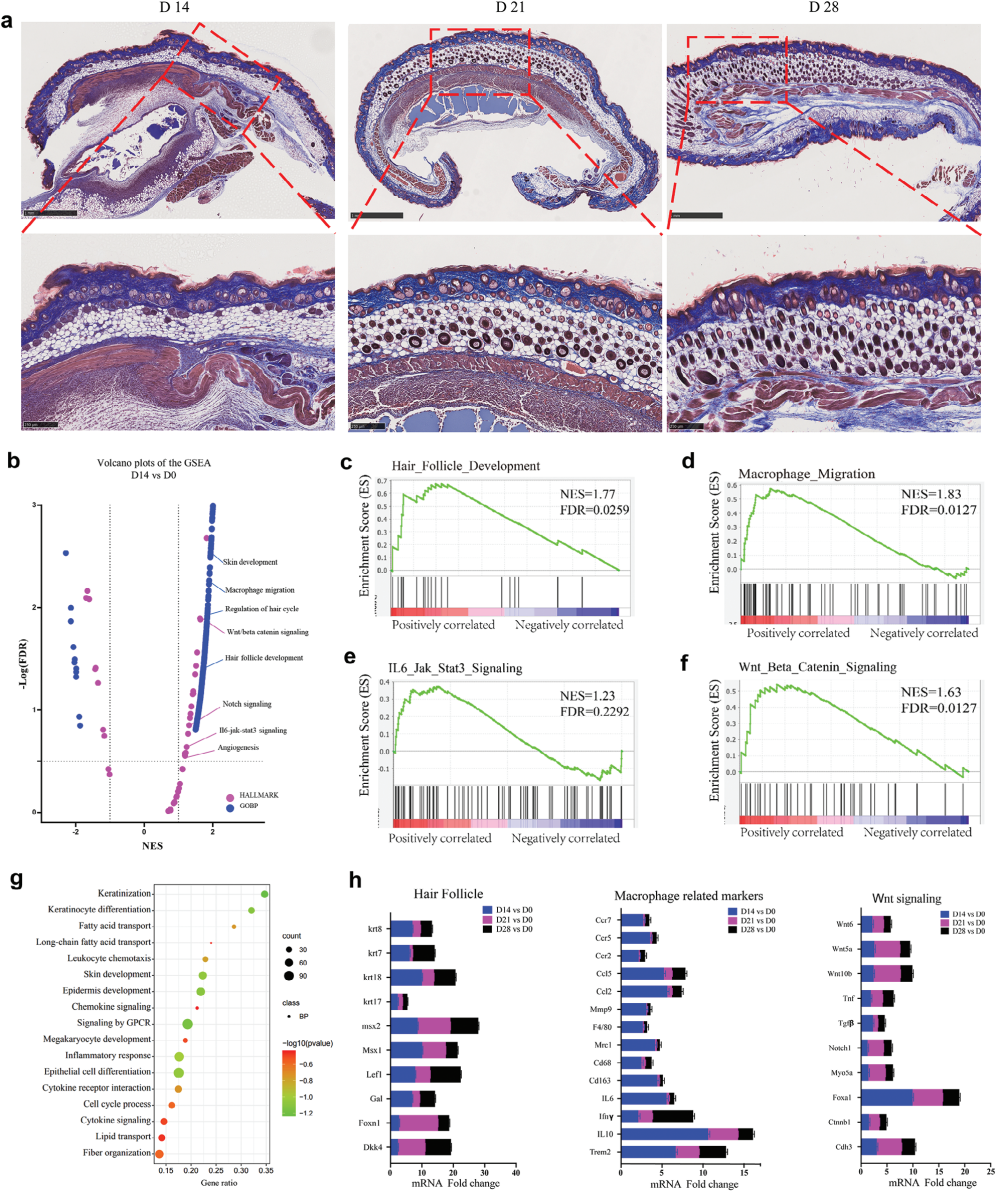
Analysis of skin tissues response to 5 mm × 5 mm × 2 mm CEWH implants. a) Masson's trichrome staining of mouse skin sections containing CEWH implants (scale bars: 1 mm; enlarged scale bars: 250 µm). Sections were collected on days 14, 21, and 28 post‐CEWH implantation. b) Volcano plots showing the gene set enrichment analysis (GSEA)‐enriched pathways in a mouse skin section with a CEWH implant, collected on day 14 post‐implantation. c–f) GSEA‐enriched signaling pathways associated with HFs development, macrophage activation, IL6/JAK/STAT3 signaling, and Wnt/*β*‐Catenin signaling in mouse skin sections with a CEWH implant (collected on day 14 post‐implantation). g) Venn diagrams illustrating the overlap of GSEA results from mouse skin sections with CEWH implants, collected at different time points post‐implantation. h) Expression levels of selected genes involved in the pathways.

Interestingly, the CEWH implant group demonstrated distinct effects (**Figure** **5**a; Figure S13, Supporting Information). Regenerated HFs appeared near the fat layer as early as day 14 and became abundant within the thickened subcutaneous adipose tissue by day 21. Even after the complete degradation of CEWH on day 28, the number and growth depth of HFs continued to increase and persist, demonstrating an ongoing regeneration process. This phenomenon suggested that CEWH effectively activated HFs toward the anagen phase and maintained this activation throughout the early growth stages.^[^ ^28^ ^]^ Additionally, in conjunction with new HFs development, the formation of new adipocytes was observed on day 21, with their number and size increasing until day 28.^[^ ^29^ ^]^

The histological analysis supported the interpretation that CEWH induced long‐lasting activation of HFs toward the early anagen phase, priming them for hair regrowth rather than maintaining them in the resting telogen phase.^[^ ^30^, ^31^ ^]^ The observed disparity in skin HFs regeneration between CEWH and EW hydrogels was likely attributed to their differing degradation rates and morphological structure. While both the EW and CEWH hydrogels demonstrate HFs regeneration ability, the components of the EW appear to be involved in initiating HFs regeneration. The extended degradation period of CEWH, which aligns with the HFs regeneration cycle, and its expanded pore structure, which facilitates long‐term cell and vascular infiltration for tissue integration, are critical for the continuous modulation of the HFs growth cycle.

To elucidate the underlying mechanism of HFs regeneration, we further analyzed the transcriptional profiles of mouse skin sections containing CEWH implants at various post‐implantation time points. Metascape analysis revealed the upregulation of HFs development and keratinization on day 14, followed by upregulation of lipid metabolism and adipogenesis on day 21, and enhanced wound healing and vasculature development on day 28 (Figure 5b; Figure S14, Supporting Information). Similarly, gene set enrichment analysis (GSEA) indicated upregulated signatures of the Wnt/*β*‐catenin and Notch signaling pathways (Figure 5c,f; Figure S15, Supporting Information), which are crucial for regulating the hair growth cycle.^[^ ^32^ ^]^ We also observed a significant upregulation of Trem2 and Ccl3, indicators of macrophage recruitment signaling (Figure 5d).^[^ ^33^ ^]^ Dermal macrophages play a crucial role in regulating major HFs regeneration pathways.^[^ ^34^ ^]^ At the onset of HFs activation (day 14), we observed upregulated expression of the IL‐6/JAK/STAT3 signaling pathway, which driven macrophage polarization from the M1 to the M2 phenotype (Figure 5e).^[^ ^35^, ^36^ ^]^ The observed temporal coordination of transcriptional regulatory factors within macrophage cell lines closely parallels changes in the Wnt/*β*‐catenin and Notch signaling pathways. This observation suggests that the M2 macrophage‐dominated immune microenvironment triggered by CEWH implantation activates HFs via the Wnt/*β*‐catenin and Notch signaling pathways. CEWH implantation was pivotal in inducing macrophage migration and polarization. This hypothesis was supported by the observation that HFs adjacent to the implant site were confined to the dermal layer and remained arrested in the telogen phase (Figure 5a). Venn diagrams further supported these observed phenotypes and signaling pathways (Figure 5g; Figure S16, Supporting Information). Our transcriptomic data, validated by qPCR data (Figure 5h; Table S1, Supporting Information), suggests a potential signaling pathway involving M2 macrophages recruited by CEWH to modulate the hair growth cycle and HFs regeneration.

Consistent with the findings from the mouse subcutaneous HFs regeneration model, visual inspection revealed significantly more vigorous hair growth at the implant site compared to neighboring skin on day 21 (Figure S17a, Supporting Information). To further confirm this observation, we performed extensive hair removal treatment to synchronize the growth cycle of all HFs. Following this treatment, the hair density and growth rate at the implant site remained demonstrably higher than in surrounding areas (Figure S17b, Supporting Information). This finding suggested a greater number of actively growing HFs in adult mice after CEWH implantation.

**Figure 6:**
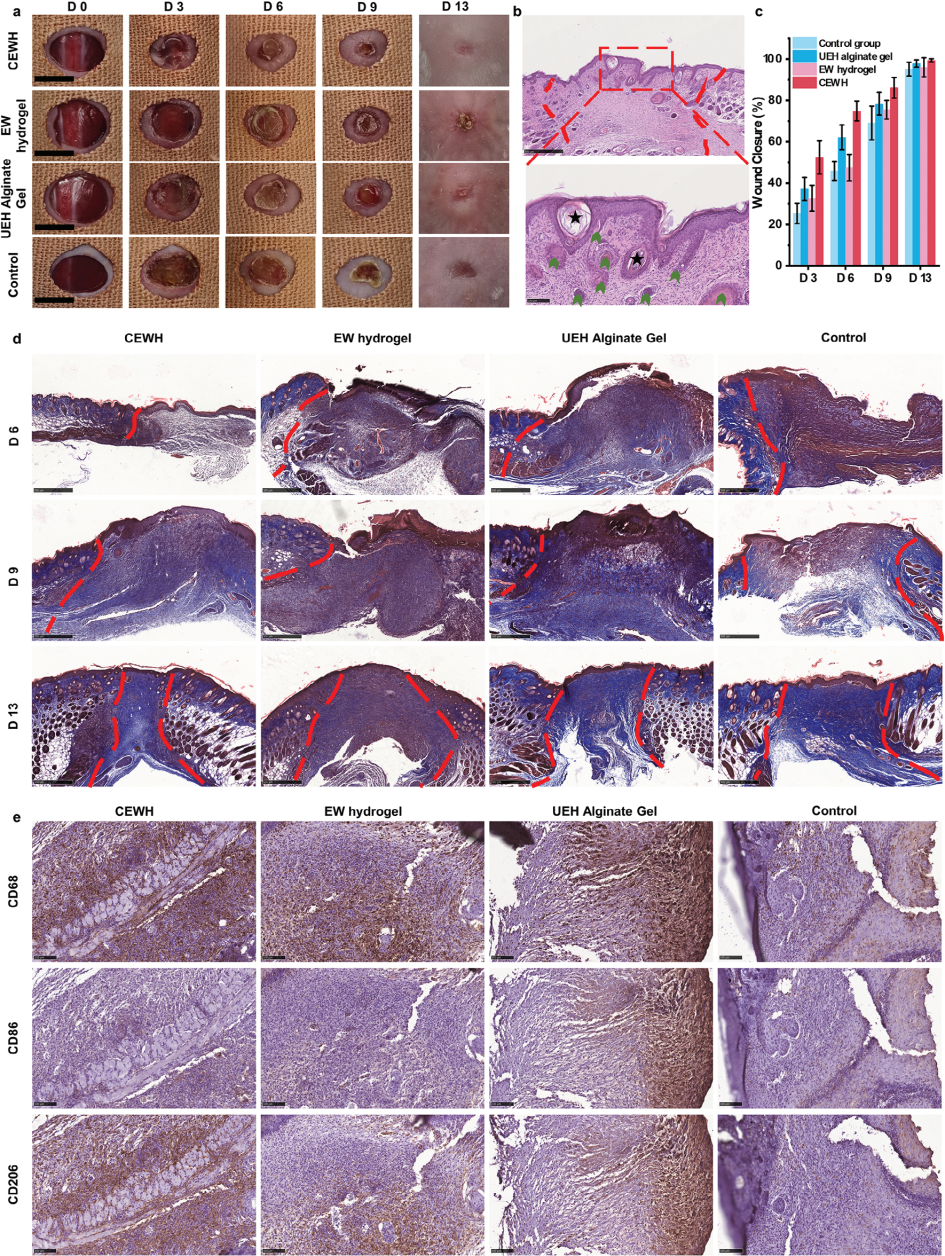
CEWH promotes wound closure and HFs regeneration. a) Representative images (scale bars: 5 mm) showing wound closure in BALB/c mice at days 3, 6, 9, and 13 post‐wounding. Four groups were investigated: CEWH‐treated group, EW hydrogel‐treated group, UEH alginate gel‐treated group, and untreated control group. Medical tape was applied around the wounds to prevent abnormal wound contraction. b) Representative images (scale bars: 500 µm) of CEWH‐treated healing wound sections on day 13 post‐wounding, stained with H&E. Asterisks indicate epidermal cysts, and arrows point to newly formed hair follicles (HFs). c) The graph depicts the wound closure rates for the four groups. d) Representative images (scale bars: 500 µm) of wound sections for the four groups on days 6, 9, and 13 post‐wounding, stained with Masson's trichrome. e) Immunohistochemistry (IHC) staining for the four groups of CD68 (macrophage marker), CD86 (M1 macrophage marker), and CD206 (M2 macrophage marker) on day 6 post‐wounding. Scale bars: 100 µm.

Given the observed ability of CEWH to support cell proliferation and recruit macrophages in vivo, we investigated its potential benefit for skin repair. Using a mouse model of excisional wound healing with BALB/c mice, we evaluated CEWH's wound‐healing performance. We established a no‐treatment control group and three experimental groups: CEWH‐treated, EW hydrogel‐treated, and UEH alginate gel‐treated (commercial wound dressings, UEH – Tianjin Jiashitang Technology Co.). Round hydrogels patches (6‐mm diameter) were placed onto the wound bed, ensuring a secure connection with wound tissue and protection from external disruptions. To further prevent abnormal wound contraction, medical tape was applied around the wounds (**Figure** **6**a). The rate of wound closure was then measured over time (Figure 6c).

The CEWH‐treated group exhibited accelerated wound closure compared to the other three groups. By day 6 post‐wounding, the CEWH group achieved 74% wound closure, whereas UEH alginate gel group, EW hydrogel group and control group showed only ≈62%, 47%, 45% closure, respectively. By day 13, wounds treated with CEWH were healed, with the healed skin appearing flat and nearly scars‐free, even showing signs of new HFs. We next examined skin histological sections to confirm the observed HFs regeneration. Consistent with the observation in Figure 6a, histological sections from the CEWH‐treated healed wounds revealed clear signs of newly formed HFs, closely related with epidermal cysts developing within the collagen matrix of the wound bed (Figure 6b).^[^ ^37^ ^]^ In contrast, the healed wounds in other three groups displayed noticeable scars and significant pigmentation.

To observe the dynamic deposition of collagen during the wound healing process, Masson's trichrome staining was performed on days 6, 9, and 13 post‐injury (Figure 6d). In this staining method, collagen fibers typically appear as blue thread‐like structures. On the 6th day post‐injury, the wound sites in all four groups were heavily infiltrated by immune cells, indicating the inflammatory phase, with no significant collagen deposition observed. By the 9th day post‐injury, the EW hydrogel and control groups still exhibited a large aggregation of immune cells, while the CEWH and UEH alginate gel groups showed the presence of collagen fibers in the wound bed. By the 13th day post‐injury, the wound beds in all four groups were nearly completely closed. The EW hydrogel group exhibited an excessive inflammatory response. The UEH alginate gel and control groups displayed scar‐like skin structures with sparse collagen fibers and noticeable cavities. In contrast, the CEWH group showed orderly collagen fiber arrangement and the presence of new HFs. This suggested that CEWH promoted collagen deposition, thereby accelerating wound healing.

Natural wound healing in adult mammals typically results in keloid scars with excessive collagen deposition and the absence of complex tissue structures such as HFs and cutaneous fat, due to intense inflammation aimed at restoring the skin barrier.^[^ ^38^ ^]^ Given that subcutaneous CEWH implantation can regulate the immune microenvironment and promote HFs regeneration by facilitating the transition from the telogen phase to the anagen phase of hair growth cycle, we hypothesized that similar mechanisms involving the Wnt/*β*‐catenin and Notch signaling pathways, mediated by M2 macrophages within a partially modulated immune environment, might be occurring during CEWH‐promoted wound healing.

To investigate whether CEWH modulates the immune response and recruits M2 macrophages during wound healing, we performed immunohistochemistry (IHC) staining on healed wound tissue sections (Figure 6e; Figure S18, Supporting Information). Compared to the commercial hydrogel group and the control group, the experimental groups treated with EW protein (including the CEWH‐treated group and the EW hydrogel‐treated group) exhibited stronger expression of CD206 (M2 macrophage marker) signal and weaker expression of CD86 (M1 macrophage marker) signal on both the 6th and 9th days post‐injury. These findings suggested that the presence of CEWH on an excisional wound could trigger the continuous and positive regulation of M2 dermal macrophages. This, in turn, could enhance HFs regeneration and accelerate wound healing with restored skin functions.

Given CEWH's excellent biocompatibility and essential nutrient content, we investigated its potential as a topical skincare dressing. A 15‐min application of CEWH increased the moisture content of hand skin and improved skin surface clarity (Figure S19a, Supporting Information). When applied to facial skin with acne, CEWH significantly reduced redness and inflammation characteristics (Figure S19b, Supporting Information). These results suggested promising applications of CEWH in daily skincare.

## 3 Conclusion

This study reported the development and application of a biocompatible EW‐derived hydrogel for promoting HFs regeneration and wound healing. CEWH was synthesized from a diluted solution of EW and CDs. This transparent, stretchable material displayed enhanced tensile strength and an enlarged pore structure. Upon heating, the EW protein chains unfolded and bonded with CDs, forming a low‐dense yet stable 3D network, thus facilitating the release of bioactive substance encapsulated within the hydrogel. Importantly, CDs acted as effective nano‐linkers, preventing extensive self‐crosslinking of the protein chains and enabling their efficient opening.

CEWH's rich protein content and large porous structure create an ideal scaffold for cell ingrowth, aggregation, and vascular penetration. This promotes excellent integration with surrounding tissues. Following subcutaneous implantation, CEWH effectively modulates the microenvironment by recruiting macrophages and promoting their continuous proliferation in the M2 phenotype via the IL‐6/STAT3 pathway for several weeks. This sustained immune microenvironment modulation effectively enhances HFs regeneration. Additionally, the slower degradation rate and improved tissue integration of CEWH contribute to long‐term regrowth.

We further demonstrated that the immune response activated by CEWH accelerated wound closure and promoted skin functional recovery in mouse models. The combination of CEWH's unique properties, its facile preparation method, and the abundance of EW as a source material suggests its promising potential as a natural biomaterial for human HFs regeneration and other tissue repair and regeneration applications.

## 4 Experimental Section

### Synthesis of CDs

Citric acid (2 g) and urea (6 g) were dissolved in 30 mL of DMSO. The solution was then heated at 160 °C for 4 h under solvothermal conditions. After cooling to room temperature, the solution was mixed with 60 mL of ethanol and centrifuged at 8000 rpm for 5 min to remove residual solvent and by‐products. The collected precipitate was redissolved in water and purified through dialysis (molecular weight cut‐off of 1000 Dalton) for 24 h. Finally, the aqueous solution from the dialysis bag was collected and freeze‐dried to obtain CDs.

### Synthesis of 1‐CDs@EW, 4‐CDs@EW, 8‐CDs@EW (CEWH), and 12‐CDs@EW

1 mL of CDs aqueous solutions with different concentrations (1, 4, 8, and 12 mg ml^−1^) were respectively added to 40 mL of a pure‐EW solution, and the mixture was diluted with pure water in a 1:1.2 dilution ratio. Subsequently, the solution was centrifuged at 10 000 rpm for 10 min, resulting in a clear supernatant solution containing EW proteins and CDs. The solution was then heated in a boiling water bath for 20 min to form transparent CDs‐crosslinked EW protein hydrogels. Based on the concentration of CDs solution added to the hydrogels, the synthesized hydrogels were named 1CDs@EW, 4CDs@EW, 8CDs@EW (CEWH), and 12CDs@EW.

### Synthesis of EW Hydrogel

The EW isolated from a raw egg was heated in a boiling water bath for 20 min, resulting in the formation of a white and opaque EW hydrogel.

### Morphology and Optical Characterization

The morphology of CDs was characterized using a Tecnai G2 F30 transmission electron microscope (FEI, USA) operated at 200 kV. UV–vis absorption spectra were measured using a V‐770 UV–vis spectrophotometer (Jasco, USA). Photoluminescence (PL) spectra were measured at room temperature using an FS5 spectrofluorometer (Edinburgh Instruments, UK). The spinnability properties of a pure‐EW solution, EW + CDs solution, CEWH and exudates were tested using a Chirascan V100 circular dichroism spectrometer. The microstructures of lyophilized hydrogels were imaged using a ZEISS Sigma scanning electron microscope (Zeiss, Germany) operated at 3 kV. An optical resolution photoacoustic microscopy (OR‐PAM) system (VIS‐50; PAOMTek, China) was used for imaging blood vessels at the implant sites.

### Rheological and Mechanical Characterization

Using the HAAKE MARSIII rheometer (Thermo Fisher), the rheological properties of EW protein hydrogels were investigated with varying concentrations of CDs through dynamic amplitude and frequency sweep experiments. To measure the yield stress of the developed hydrogels, amplitude sweep experiments were conducted at a constant frequency of 5 Hz. Additionally, dynamic frequency sweep experiments were performed at frequencies ranging from 1 to 35 Hz for all hydrogels with different CDs concentrations to explore the relationship between the gel features and frequency variations.

The mechanical properties of pure EW hydrogel, 4CDs@EW, CEWH, and 12CDs@EW were evaluated using a tensile testing machine (MTS Exceed E44, MTS Systems, USA) equipped with a 50 N transducer. Dumbbell‐shaped specimens with the initial dimensions of 16.0 mm × 4.0 mm × 1.0 mm underwent tensile tests at a constant rate of 20 mm min^−1^ at room temperature. Tensile stress (σ) and strain (ε) were continuously recorded. Stress was calculated by dividing the applied force by the initial cross‐sectional area, and strain was determined by dividing the gauge length change by the initial gauge length.

### Characterization of Adhesion Properties of CEWH

The lap shear test of CEWH was conducted using the MTS Exceed E44 mechanical tester to characterize its adhesion properties. A hydrogel patch measuring 16.0 mm × 4.0 mm × 1.0 mm was adhered to pig skin tissue and pressed for 2 min to ensure proper adhesion. The test was performed at a constant tensile speed of 20 mm min^−1^ to evaluate the hydrogel's adhesion to skin tissues.

### Biocompatibility and Cytotoxicity Assessment

Mouse fibroblast cell line NCTC clone 929 (L929) and mouse embryonic fibroblast cell line NIH3T3 were used to assess the cytotoxicity of CEWH exudate. Cells were seeded at a density of 10 000 cells per well in 96‐well plates and cultured overnight. The cell culture medium was then replaced with 100 µL fresh medium containing different concentrations of hydrogel PBS exudates. Different concentrations of this hydrogel PBS exudates solution were prepared by adjusting the volume ratio of the hydrogel exudate to the cell culture medium, with specific ratios as follows: I) 11 µL of hydrogel exudate in 89 µL of cell culture medium; II) 20 µL in 80 µL; III) 33 µL in 67 µL; IV) 50 µL in 50 µL. The control group for the cytotoxicity experiment consisted of an equal volume of PBS solution replacing the hydrogel exudate. Cell viability was examined using the cell counting kit‐8 (CCK‐8, Dojindo, Tokyo, Japan) assay on days 1 and 2 after co‐culturing.

To prevent interference from the inherent fluorescence of CDs, the medium was removed before the toxicity assay and the cells were washed twice with fresh medium. Then, 100 µL of fresh medium containing 10 µL of CCK‐8 was added to each well, and the cells were incubated for another 3–4 h. The absorbance at 450 nm was recorded using a Varioskan LUX Multimode Microplate Reader (Thermo Fisher Scientific). Each experiment was performed in triplicate wells in parallel.

MDA‐MB‐231 cells were used to assess the biocompatibility of CEWH. CEWH was first placed in an empty six‐well plate with the stripped surface up. 0.5 mL of a density of 10000 MDA‐MB‐231 cells were slowly dripped onto the stripped surface of CEWH and left for ≈10 min to allow the cells to adhere to the stripped surface of CEWH as much as possible. Subsequently, a pipette gun was used to slowly add culture medium along the well walls of the six‐well plate, and it was again left to stand for ≈10 min. The six‐well plates were carefully placed at 37 °C and 5% CO_2_ for 24, 48, and 72 h. An quantitative image‐based cytometry assay was performed by capturing images of the CEWH surface using an IN Cell Analyzer 2000 instrument (GE Healthcare, USA).

### In Vitro Two‐Photon Fluorescence Imaging (TPF)

The cell culture procedure was the same as the cytotoxicity assessment above, but green fluorescence‐labeled Hela (Hela‐GFP) cells were used instead of MDA‐MB‐231 cells to meet the requirements for two‐photon imaging. Hela cells’ GFP fluorescence can be specifically excited with a 960‐nm femtosecond laser. The CEWH seeded with Hela‐GFP cells was placed on a culture dish suitable for confocal microscopy to perform in vitro TPF.

For TPF imaging, a wavelength‐tunable (700–1300 nm) near‐infrared femtosecond laser (InSight X3; Spectra‐Physics, USA) was used as the light source. This minimizes photodamage to cells and allows for deeper tissue penetration. Images were acquired using an inverted multiphoton microscope (A1MP + Eclipse Ti‐2E; Nikon Instruments, Japan) equipped with a high‐resolution 40 × water‐immersion objective with a numerical aperture (NA) of 1.15. The excited TPF and second harmonic generation (SHG) signals were collected using the same objective, reflected by multiphoton dichroic beam splitters, and detected by four photomultiplier tubes (PMTs).

### Animal Experiments

Animal experiments were approved by the Animal Ethics Committee of the University of Macau (UMAEC‐037‐2015 and UMARE‐021‐2022). Mice were housed in a specific‐pathogen‐free (SPF) facility with a 12‐h light/dark cycle. The temperature in the facility was maintained at 23–25 °C.

### In Vivo Implantation

Ten‐week‐old female BALB/c mice (n = 10), weighting between 20 and 25 grams, were anesthetized using intraperitoneal injections of Avertin. The back skin of the mice was shaved for hair removal. A linear wound ≈6 mm in length was then created using surgical scissors. Next, a curved tweezer was inserted subcutaneously to separate the tissue membrane from the back skin, creating a pocket for the implantation. The EW hydrogel patch or CEWH patch (both 5 mm × 5 mm × 2 mm) were then inserted into the subcutaneous space on the back of the mice. Finally, the wound was sealed with 3 m tissue adhesive. After awakening and recovery, the mice were returned to the SPF facility for monitoring.

### In Vivo NIR Fluorescence Imaging (NIRF)

To monitor the in vivo degradation rates of subcutaneously implanted CEWH patches, female BALB/c mice underwent in vivo NIRF imaging. An iXon Life 888 electron multiplying CCD camera (Oxford Instruments, UK) equipped with a 610‐nm long‐pass optical filter was used to perform real‐time in vivo NIRF with a 589‐nm excitation wavelength. NIRF images were collected at various time points.

### In Vivo TPF

All surgical procedures were performed under aseptic conditions within a class II biosafety cabinet. A 3 mm × 3 mm × 1 mm CEWH patch was implanted into the ear of LysM‐Cre‐mT/mG mice (*n* = 5).^[^ ^26^ ^]^ The ear was disinfected with a 75% ethanol solution. Sterile saline (50–100 µL) was subcutaneously injected into the ear using a 1‐mL syringe to create a pocket. A small incision was made with micro dissecting scissors. Curved forceps were used to widen the pocket and gently separate the ear skin. The CEWH patch was inserted into the pocket, and the incision site was disinfected with alcohol. After awakening and recovery, the mouse was returned to the SPF facility.

On days 5, 12, and 19 after CEWH implantation, the activity of macrophages in the ear tissue of LysM‐Cre‐mT/mG mice was monitored using real‐time in vivo TPF microscopy.^[^ ^26^ ^]^ Briefly, the mice were anesthetized with a 4% solution of 2,2,2‐tribromoethanol (Merck, Germany) injected intraperitoneally. Their ears were then secured on slides with transparent adhesive tape for imaging.

A wavelength tunable (700–1300 nm) near‐infrared femtosecond laser (InSight X3; Spectra‐Physics, USA) served as the light source for TPF. Images were captured using an inverted multiphoton microscope equipped with a high‐resolution 40 × water‐immersion objective with a numerical aperture (NA) of 1.15.

The mice, secured on slides with tape, were positioned on the motorized stage of the inverted multiphoton microscope. Using a 960‐nm laser pulse for excitation, the microscope captured TPF images of GFP‐labeled macrophages alongside SHG images of collagen fibers. To visualize blood vessels, separate TPF images were acquired using a 1100‐nm laser pulse for excitation.

### RNA Isolation and Quantitative Real‐Time PCR (qPCR) Analysis

Total RNA was isolated from skin tissue with TRIzol reagent (Thermo Fisher Scientific, Waltham, MA) according to the manufacturer's instructions. Briefly, a small piece of skin tissue (50–100 mg) was cut and placed into a tube with ceramic beads, and 1 mL of TRIzol was added. Put the tube for homogenize in Precellys Evolution homogenizer (30 s on, 30 s off, 2 min). Next, 200 µL of chloroform was added, shaken for 15 s (Note: do not vortex), and incubated for 10–15 min at room temperature (RT). The mixture was then centrifuged at 15 000 rpm for 15–20 min at 4 °C. The aqueous upper phase was transferred to a new tube. Isopropanol (70% of the aqueous phase or 1/2 TRIzol volume) was added and incubated for 10 min at RT. Spin max, 15 000 rmp, 10–15 min, 4 °C. The pellet was washed with 70% ethanol (EtOH), briefly vortexed, and dissolved in 100 µL of filtered or DEPC‐treated RNase‐free water (H_2_O). Reverse transcription was performed using a QuantiTect reverse transcription kit (205 313; Qiagen, Hilden, Germany). cDNA synthesis was carried out with the QuantiTect Reverse Transcription Kit (Qiagen) using 1 µg of total RNA. RT‐PCR was performed with FastStart Universal SYBR Green Master (4 913 850 001; Roche Diagnostics, Basel, Switzerland) in a QuantStudio 7 Flex real‐time PCR system (Thermo Fisher Scientific, Waltham, MA). Relative quantitation was achieved by normalization to 18S. The primers used for RT‐PCR are listed in Table S1 (Supporting Information).

### Mouse Models of Wound Healing

Four groups of 10‐week‐old female BALB/c mice (n = 10) were anesthetized with intraperitoneal Avertin injections. After pinching the skin between the shoulder blades, each mouse was positioned on its side. A sterile 6‐mm punch biopsy scalpel was used to create clean, symmetrical, full‐thickness excisional wounds on both sides of the dorsal midline. Medical tape was applied around the wounds to prevent abnormal wound contraction. Circular CEWHs, EW hydrogels, and UEH alginate gel (commercial wound dressings, UEH – Tianjin Jiashitang Technology Co.) with a diameter of 7 mm were randomly added to the wound bed on each mouse to minimize variability, respectively. Finally, the wounds were covered with self‐adhesive elastic bandages (Vetrap; 3 m, USA). These dressings were changed on days 2, 4, 6, and 9 post‐injury. Throughout the experiment (days 0, 3, 6, 9, and 13), mice were anesthetized and photographed with a Nikon D7500 digital camera. On days 6, 9, and 13, wound tissue was excised and processed for future analysis.

### Wound Closure Evaluation

Wound closure evaluation was conducted by capturing images of each wound using a high‐resolution D7500 camera (Nikon Instruments, Japan). The closure rates were determined through pixel‐area comparisons. ImageJ software was used to quantify wound closure by analyzing pixel area changes.

### Histological Analysis

Skin tissues (≈8 µm thick) were suspended in cold PBS, centrifuged, and fixed in a 4% paraformaldehyde solution for 30 min. The tissues were then dehydrated and embedded in paraffin. Sections of 5 µm thickness were prepared from the paraffin‐embedded tissues and subjected to H&E staining, Masson's trichrome staining and immunohistochemistry (IHC) for histopathological assessment.

## Conflict of Interest

The authors declare no conflict of interest.

## Author Contributions

S.Q. designed the project and was involved in every step of the investigation. C.X. Deng led the biomedical research. T.‐M.L. led the bioimaging research. Z.K.T. supervised the project. J.W. focused on CEWH synthesis and characterizations, and participated in all investigations, including cell and animal experiments. J.H.L. focused on animal experiments, skin tissue staining and RNA sequencing analysis. M.L. focused on two‐photon fluorescence imaging experiments. A. Zhang contributed to the RNA sequencing analysis. X.L. and Z.Y. contributed to the in vivo photoacoustic imaging. Senio and C.W. contributed to the cytotoxicity test. Y.L. participated in CEWH synthesis. G.C. participated on the analysis of skin tissue sections.

## Supporting information



Supporting Information

Supplemental Video 1

Supplemental Video 2

Supplemental Video 3

Supplemental Table 1

## Data Availability

The data that support the findings of this study are available from the corresponding author upon reasonable request.
