# Supplementary material for: Carbon Dots Crosslinked Egg White Hydrogel for Tissue Engineering
Source: Adv Sci (Weinh). 2024 Sep 20;11(43):2404702. doi: 10.1002/advs.202404702 (PMC11578375; doi:10.1002/advs.202404702)
Supplement: Supplementary file 1 — Supporting Information [file ADVS-11-2404702-s005.docx]

Supplementary Information

**Carbon Dots Crosslinked Egg White Hydrogel for Tissue Engineering**

Jun Wu, Josh Haipeng Lei, Moxin Li, Aiping Zhang, Yuan Li, Xiao Liang, Senio Campos de Souza, Zhen Yuan, Chunming Wang, Guokai Chen, Tzu-Ming Liu^*^, Chu-Xia Deng^*^, Zikang Tang^*^, and Songnan Qu^*^

Table of Contents

[Figure S1 3](#_Toc174642166)

[Figure S2 4](#_Toc174642167)

[Figure S3 5](#_Toc174642168)

[Figure S4 6](#_Toc174642169)

[Figure S5 7](#_Toc174642170)

[Figure S6 7](#_Toc174642171)

[Figure S7 7](#_Toc174642172)

[Figure S8 8](#_Toc174642173)

[Figure S9 8](#_Toc174642174)

[Figure S10 9](#_Toc174642175)

[Figure S11 9](#_Toc174642176)

[Figure S12 10](#_Toc174642177)

[Figure S13 11](#_Toc174642178)

[Figure S14 11](#_Toc174642179)

[Figure S15 12](#_Toc174642180)

[Figure S17 14](#_Toc174642181)

[Figure S18 15](#_Toc174642182)

[Figure S19 16](#_Toc174642183)

[Figure S20 17](#_Toc174642184)


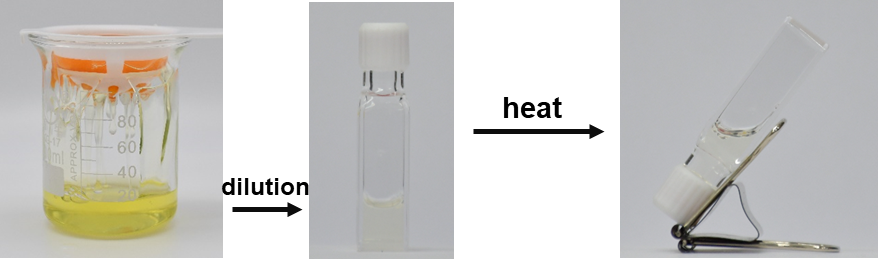


Figure S1 The reaction process of the pure EW solution under the same dilution and heating conditions used for the synthesis of CEWH.


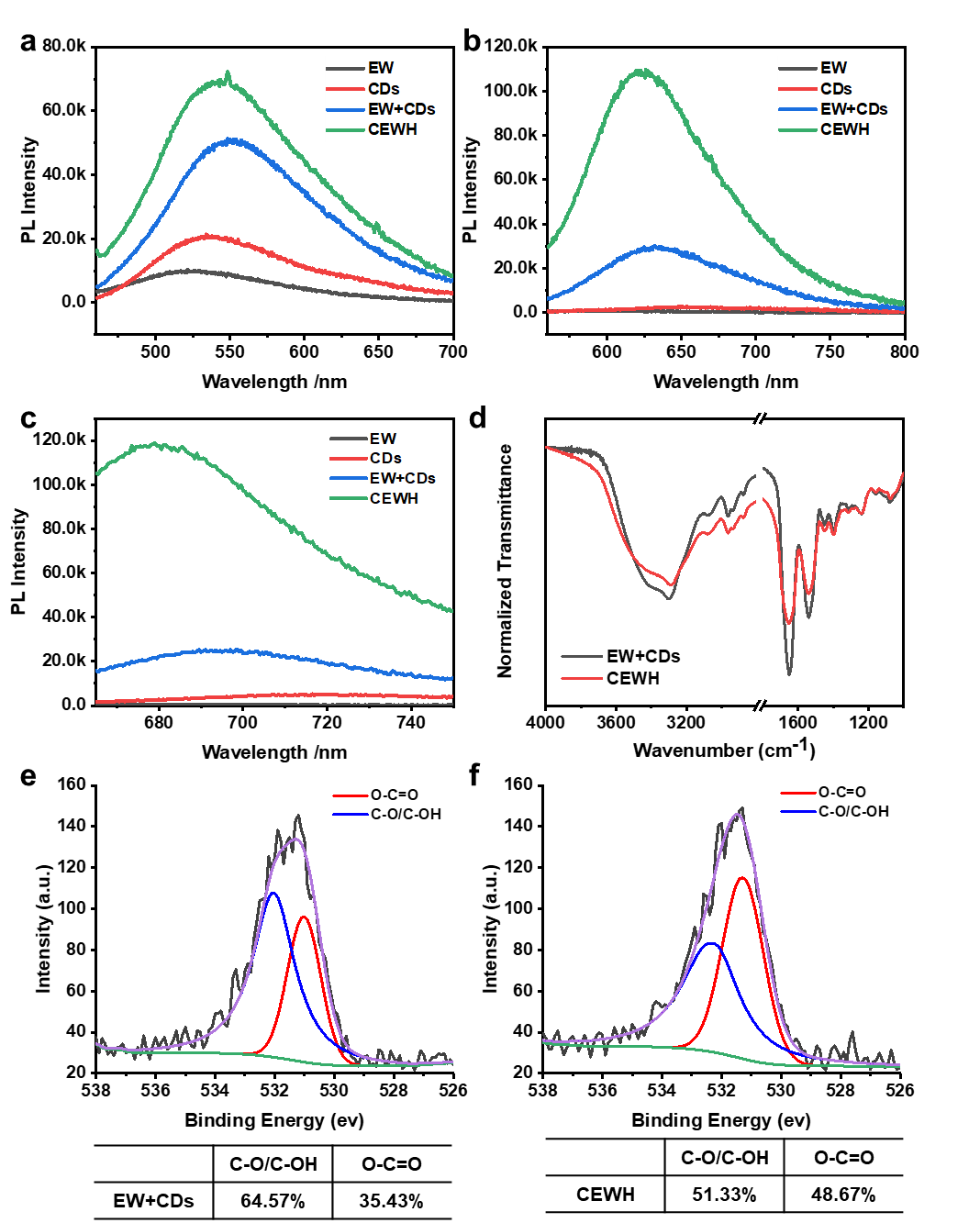


Figure S2 (a-c) The PL emission spectra of dilute EW aqueous solution (EW), CDs solution (CDs), EW+CDs solution and CEWH. (d) The measured Fourier transform infrared spectra (FT-IR) of EW+CDs and CEWH xerogel. (e, f) High-resolution O 1s XPS spectra and the specific contents of O-related chemical bonds of EW+CDs and CEWH, respectively.


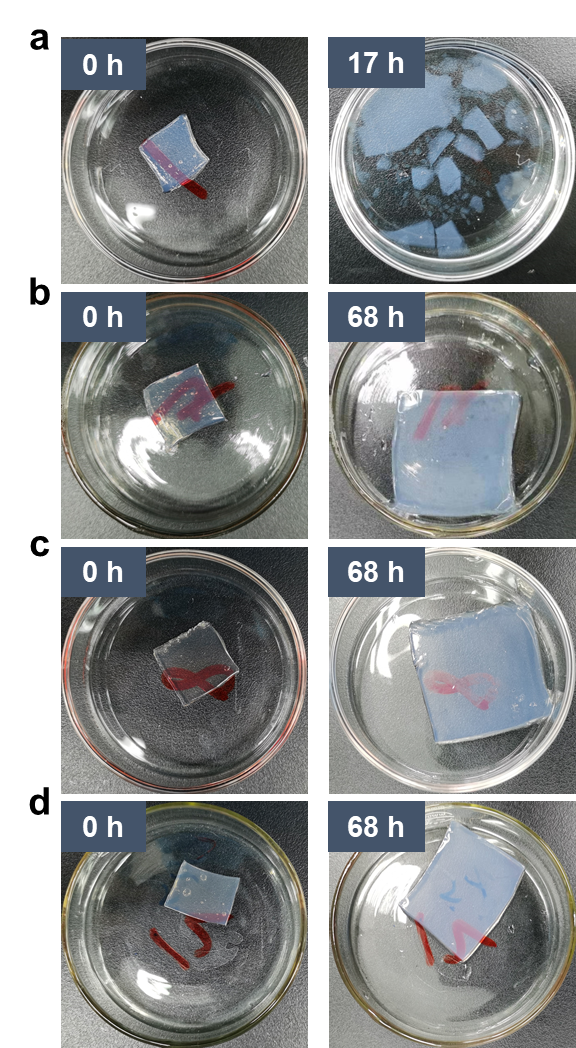


Figure S3 Images of (a) 1CDs@EW, (b) 4CDs@EW, (c) 8CDs@EW and (d) 12CDs@EW taken at the initial time and after being immersed in water for 17 h, 68 h, 68 h and 68 h, respectively.


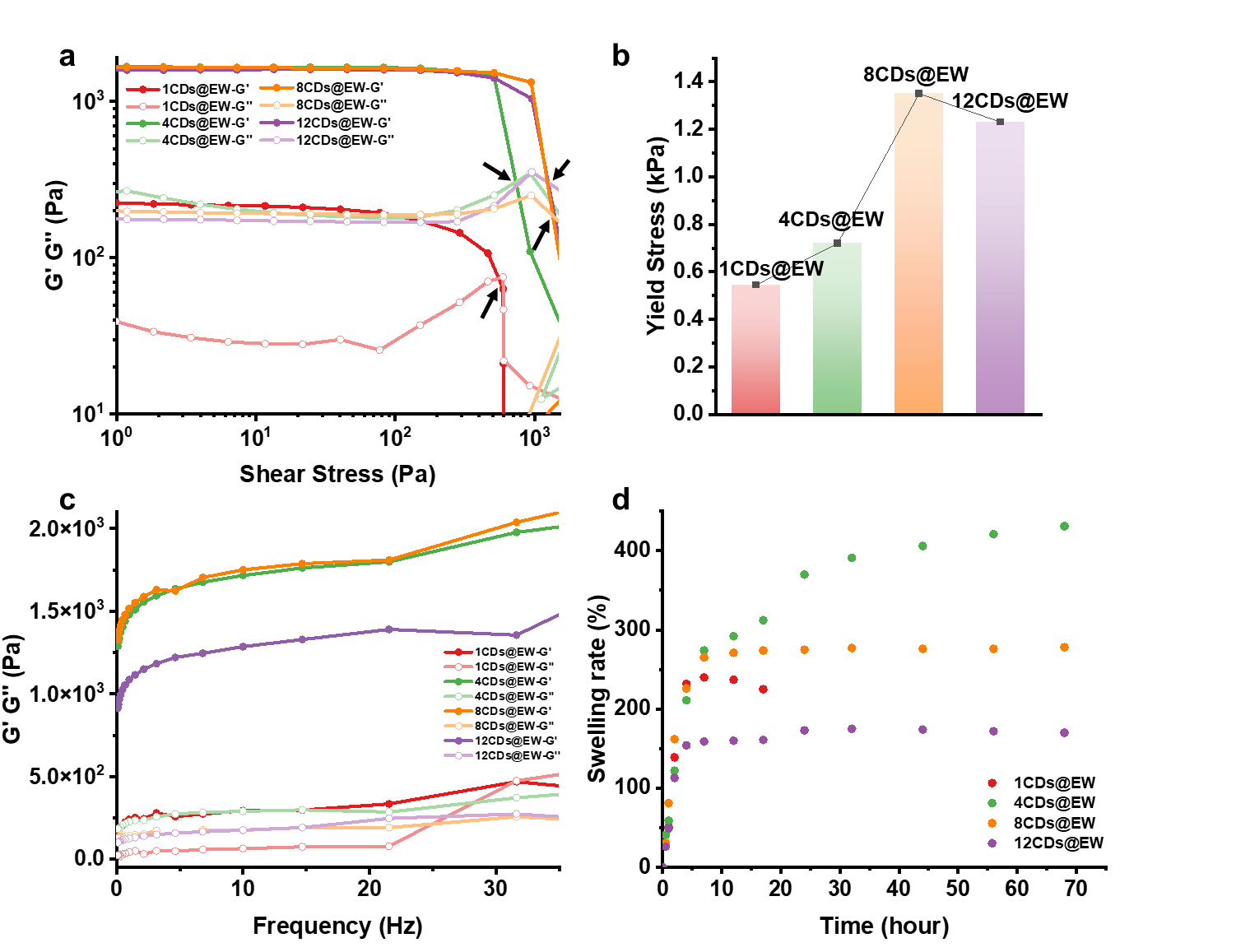


Figure S4 Rheological behavior of the four hydrogels: (a) amplitude sweep (the arrows points to where G' intersects G''), (b) yield stress, (c) frequency sweep. (d) Swelling rates of hydrogels in pure water. The swelling time was prolonged to 68 h.


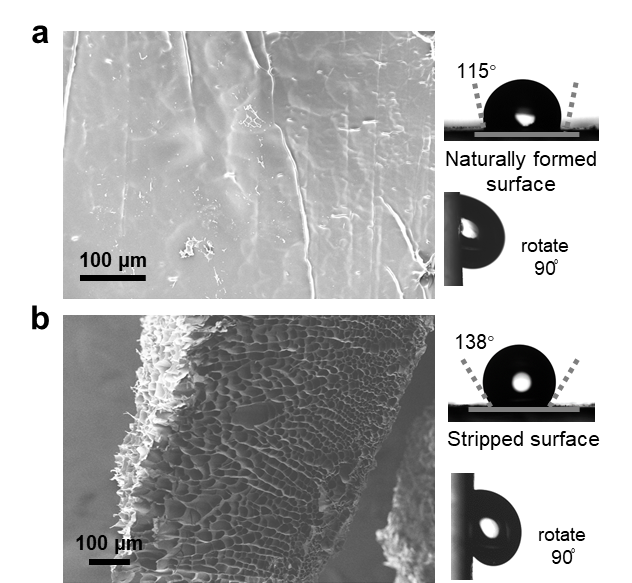


Figure S5 (a) The microstructure of the freely formed surface of CEWH and its corresponding hydrophobic contact angle. (b) The microstructure of the stripped surface of CEWH and its corresponding hydrophobic contact angle.


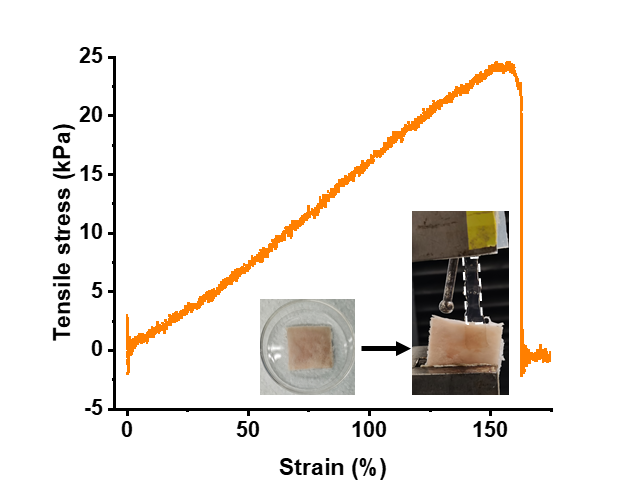


Figure S6 Representative tensile stress–strain curves characterizing the adhesive property of CEWH (8CDs@EW). CEWH patch measuring 16.0 mm × 4.0 mm × 1.0 mm was adhered to pig skin tissue to perform the lap shear test. The inserted figure shows the actual experimental setup.


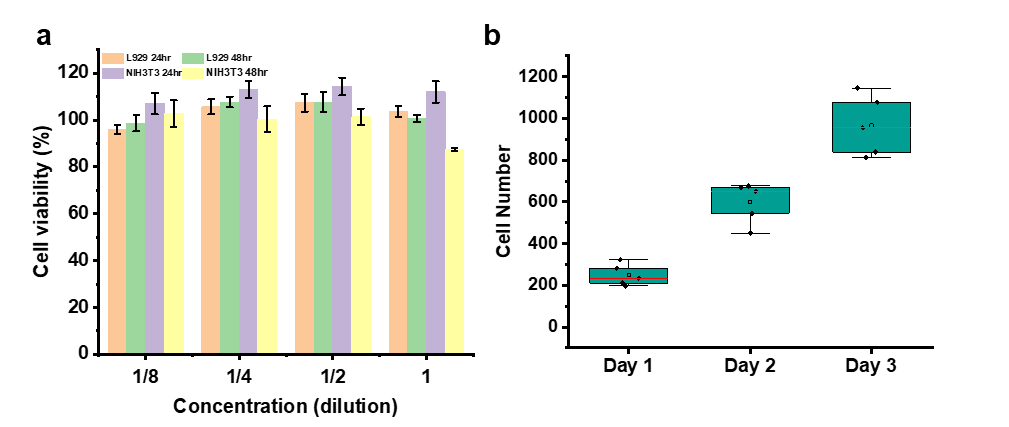


Figure S7 (a) Cytotoxicity assessment of the CEWH exudate, examined using L929 and NIH3T3 cells. N = 3. (b) The relationship between the counted number of MDA-MB-231 cells and cells incubation time. N = 5.


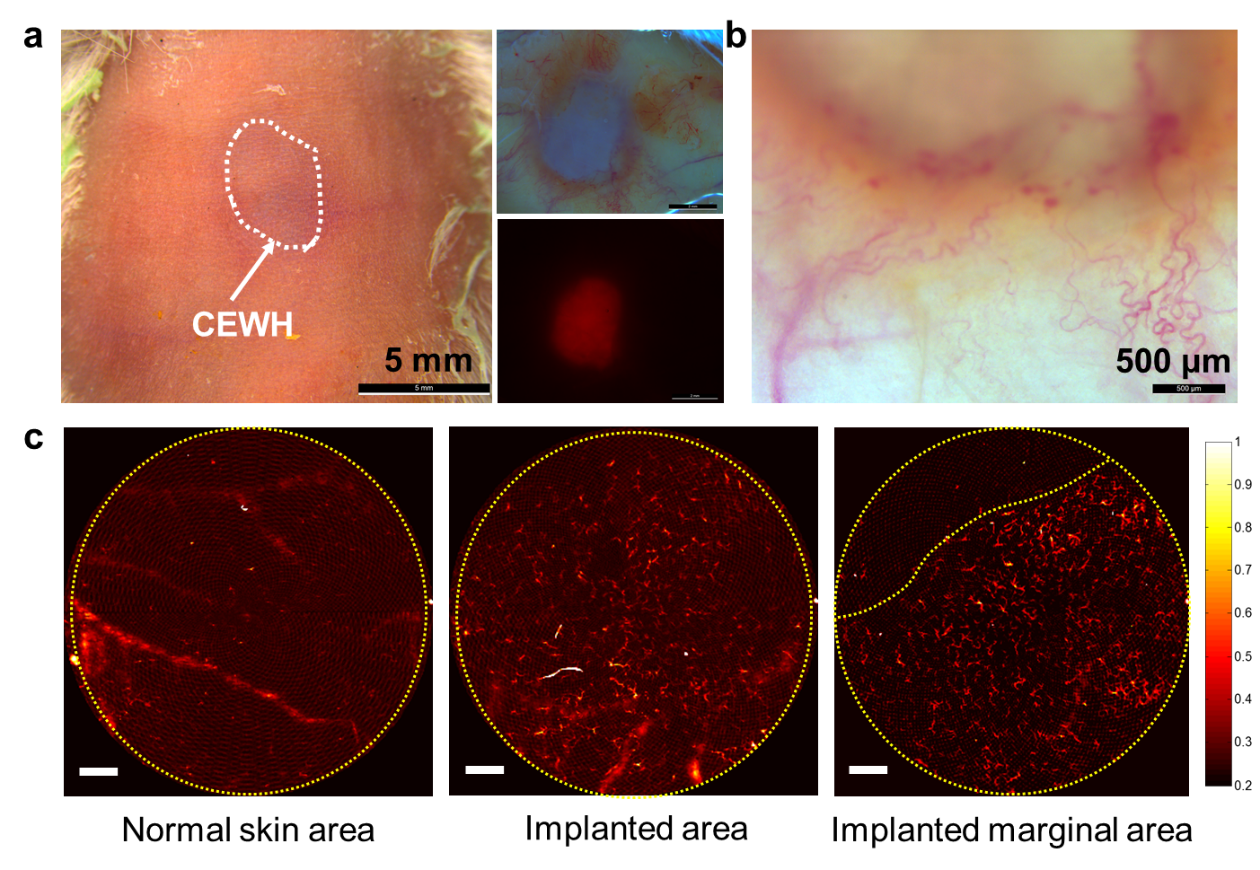


Figure S8 (a) Bright-field and fluorescence stereo microscope images of the mice skin in the CEWH implantation area. (b) A partially magnified image of the excised mouse skin with an CEWH implant. Both (a) and (b) were observed under a stereomicroscope (Body Model: Leica MZ10 F; Camera Model: Leica DFC450 C). (c) In vivo photoacoustic images of different implantation skin areas (normal skin area, implanted area, and implanted marginal area) under 532 nm excitation. Scale bars: 1 mm. All images were captured on the 21st day post-CEWH implantation.


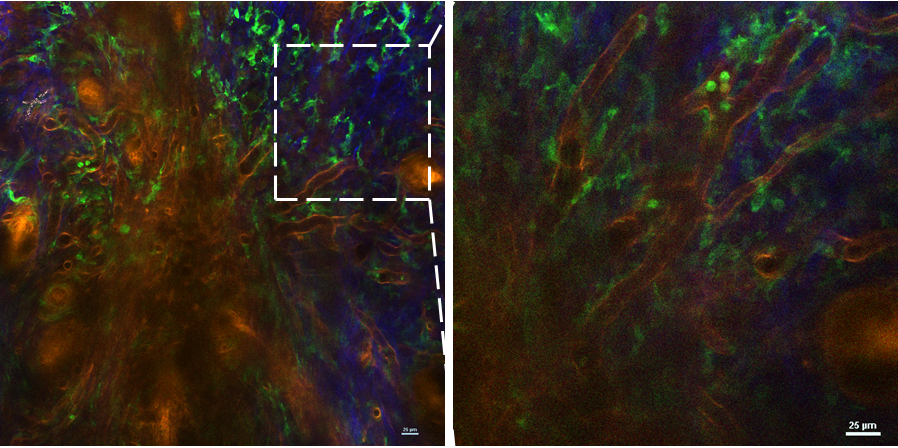


Figure S9 TPF merged images of the implant site in the ear of a LysM-Cre-mT/mG mouse on the 19th day after CEWH implantation. The images were captured in the marginal regions of the implantation site. Scale bar: 50 μm.


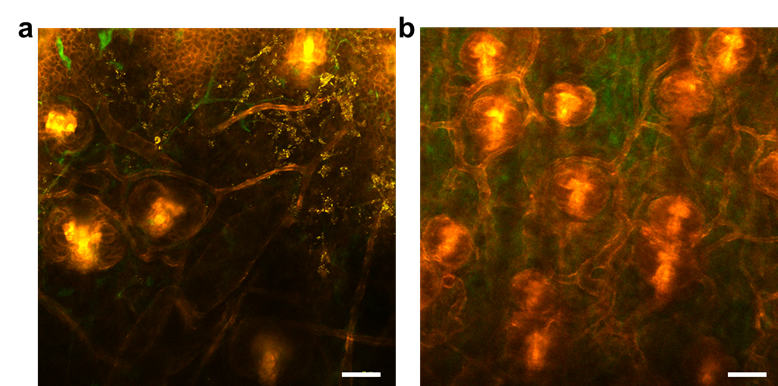


Figure S10 TPF merged images of a mouse ear. (a) Normal ear area without treatment; (b) Ear area with a subcutaneous CEWH implant. The images were collected on the day 7 after the implantation. Two femtosecond lasers with distinct wavelengths were used for excitation: 960 nm for the green channel and 1100 nm for the red channel. Scale bar: 50μm.


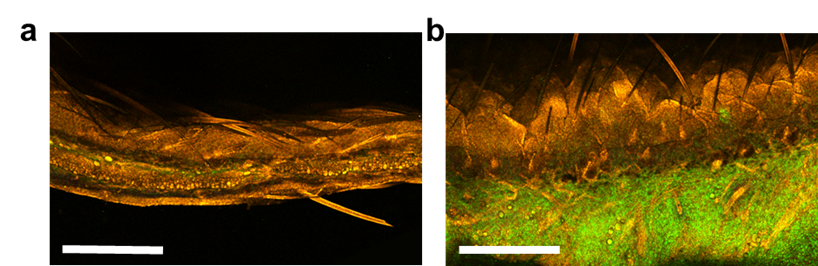


Figure S11 TPF images of frozen tissue section from a mouse ear. (a) Normal ear area ; (b) Area containing a subcutaneous CEWH implant. The section was collected on the day 12 after the implantation. Two femtosecond lasers with distinct wavelengths were used for excitation: 960 nm for the green channel and 1100 nm for the red channel. Scale bar: 500μm.


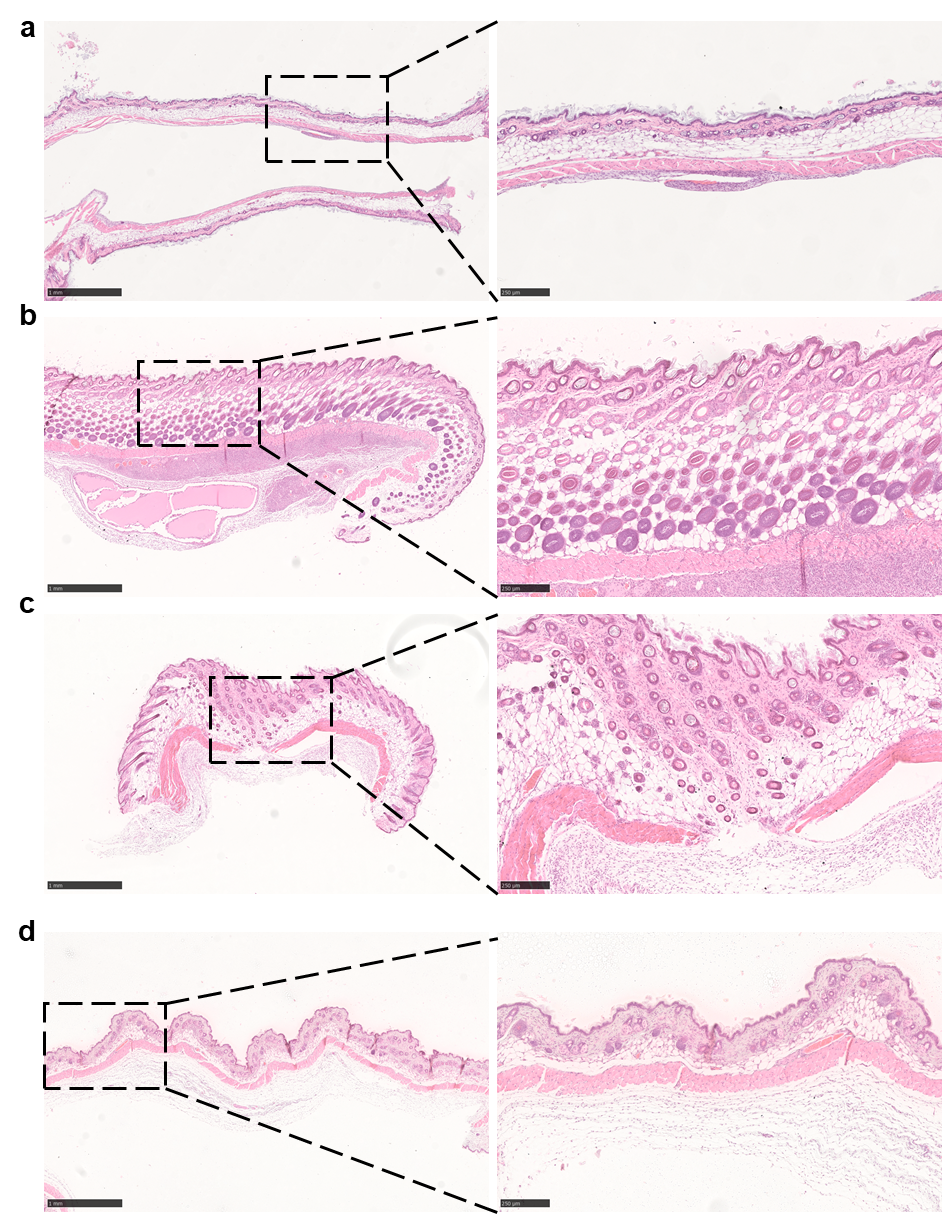


Figure S12 Hematoxylin-eosin staining of mouse skin sections from (a) the untreated group, (b) EW hydrogel-implantation group on the 14th day post-implantation, (c) EW hydrogel-implantation group on the 21th day post-implantation, and (d) EW hydrogel-implantation group on the 28th day post-implantation. Scale bars: 1 mm; Enlarged scale bars: 250 μm.


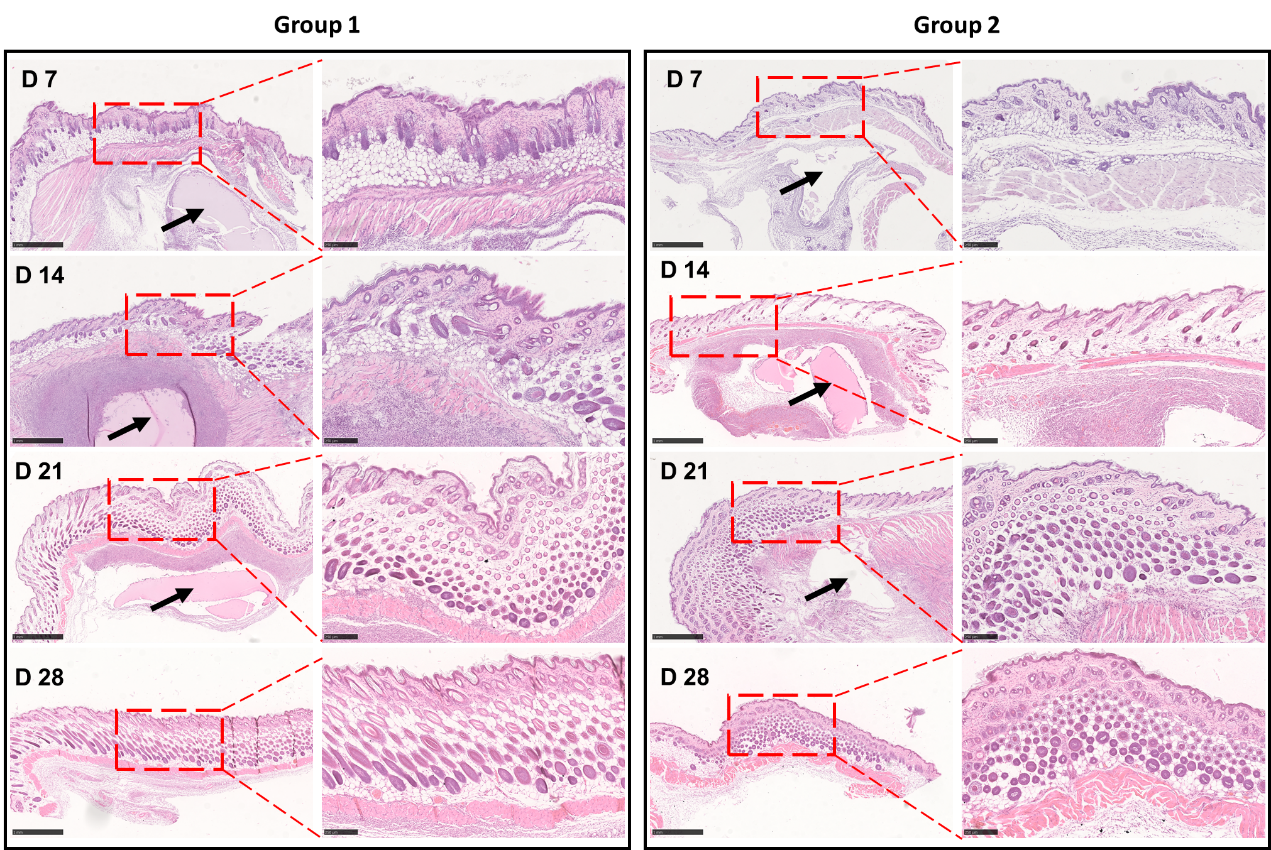


Figure S13 H&E staining of mouse skin sections containing CEWH implants (scale bars: 1 mm; enlarged scale bars: 250 μm). Sections were collected on days 7, 14, 21, and 28 post-CEWH implantation. The arrow points to the subcutaneous implant CEWH.


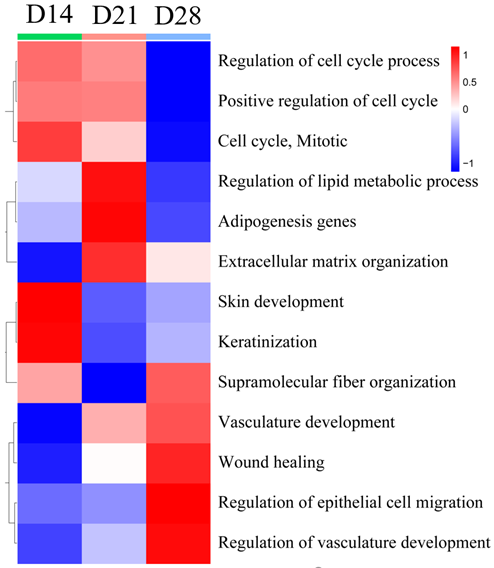


Figure S14 A heatmap representing the differential pathway enrichment in the mouse skin sections at significant time points. The columns, from left to right, represent 7th day post-implantation vs 0 day, 21st day post-implantation vs 0 day, and 28th day post-implantation vs 0 day.


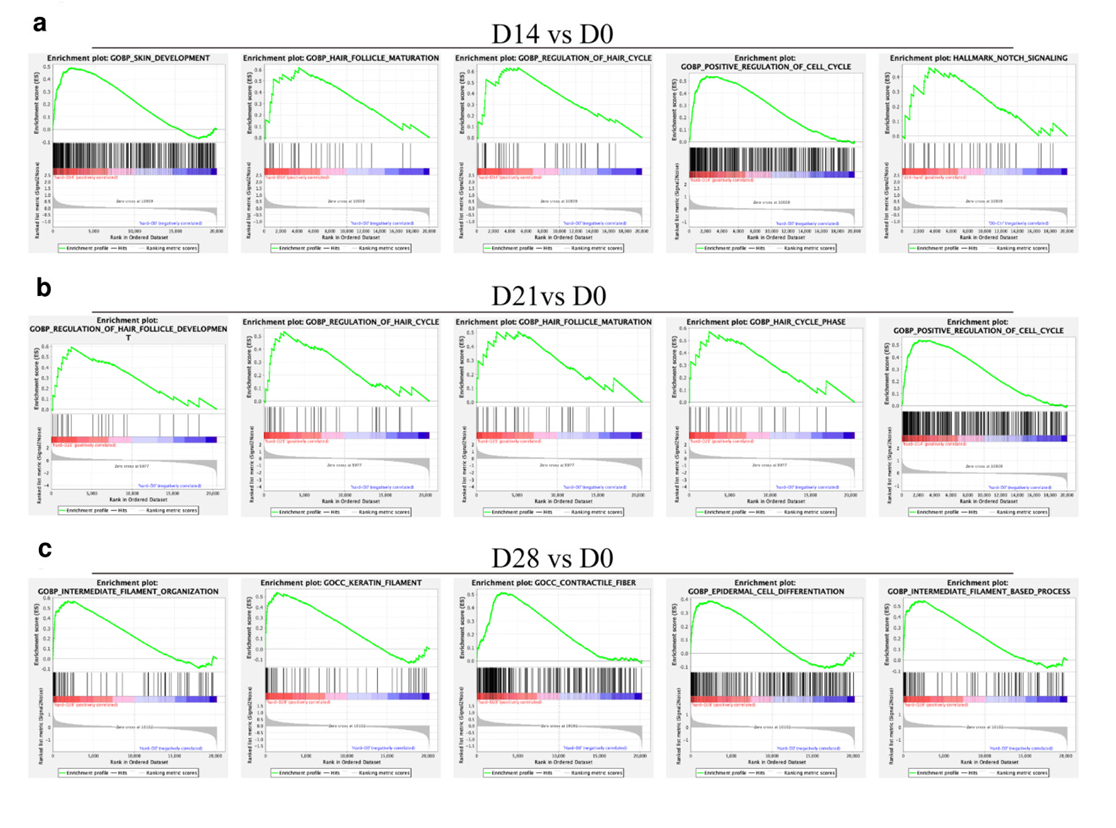


Figure S15 GSEA-enriched signaling pathways depicting the differentially expressed genes (DEGs) identified between the CEWH-implantation group and untreated groups.


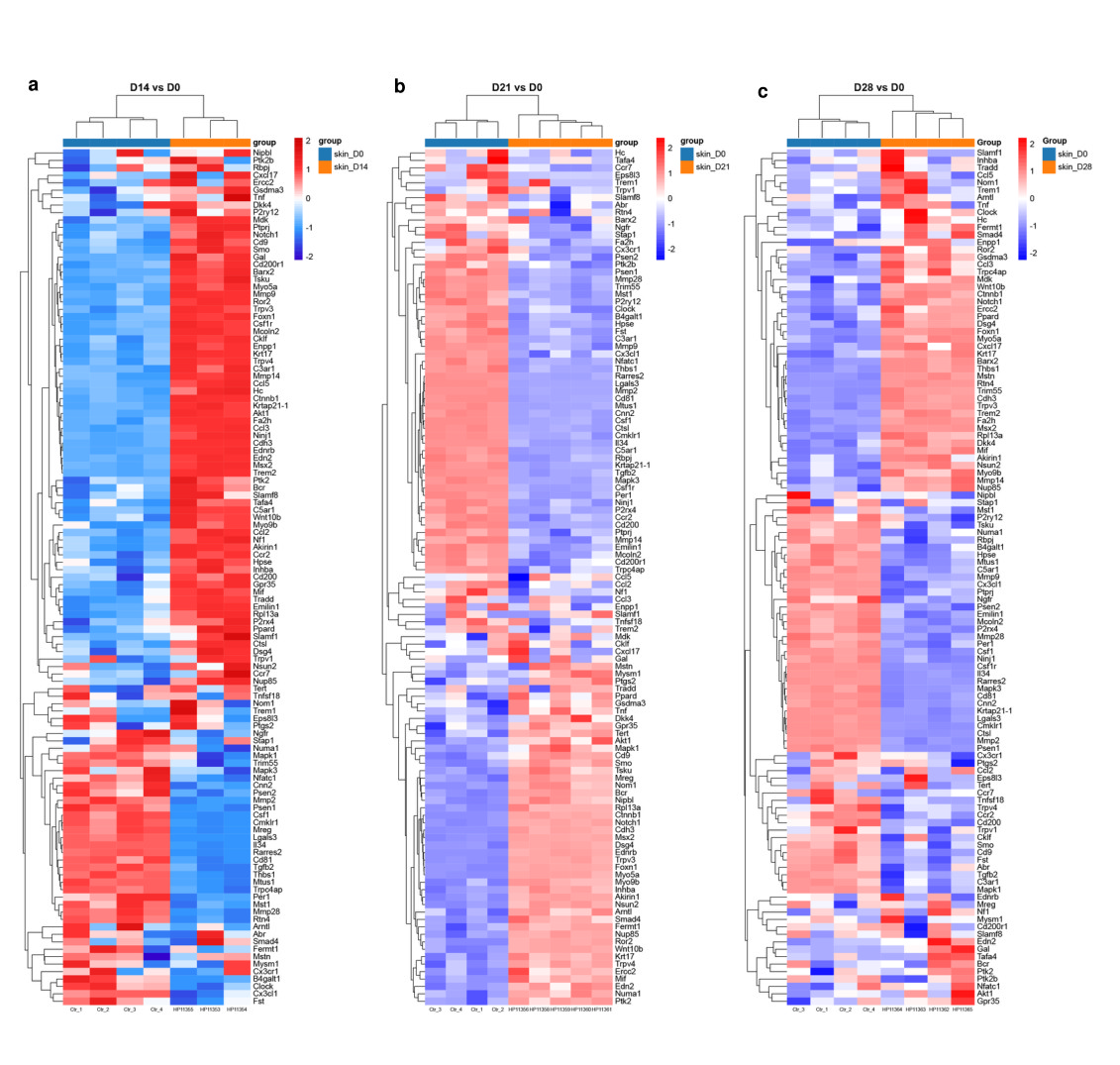
Figure S16 Heatmap of differentially expressed genes (DEGs) in mouse skin containing the CEWH implant compared to untreated-group skin, with n = 4 biologically independent samples. The heatmap shows the dynamic transcripts.


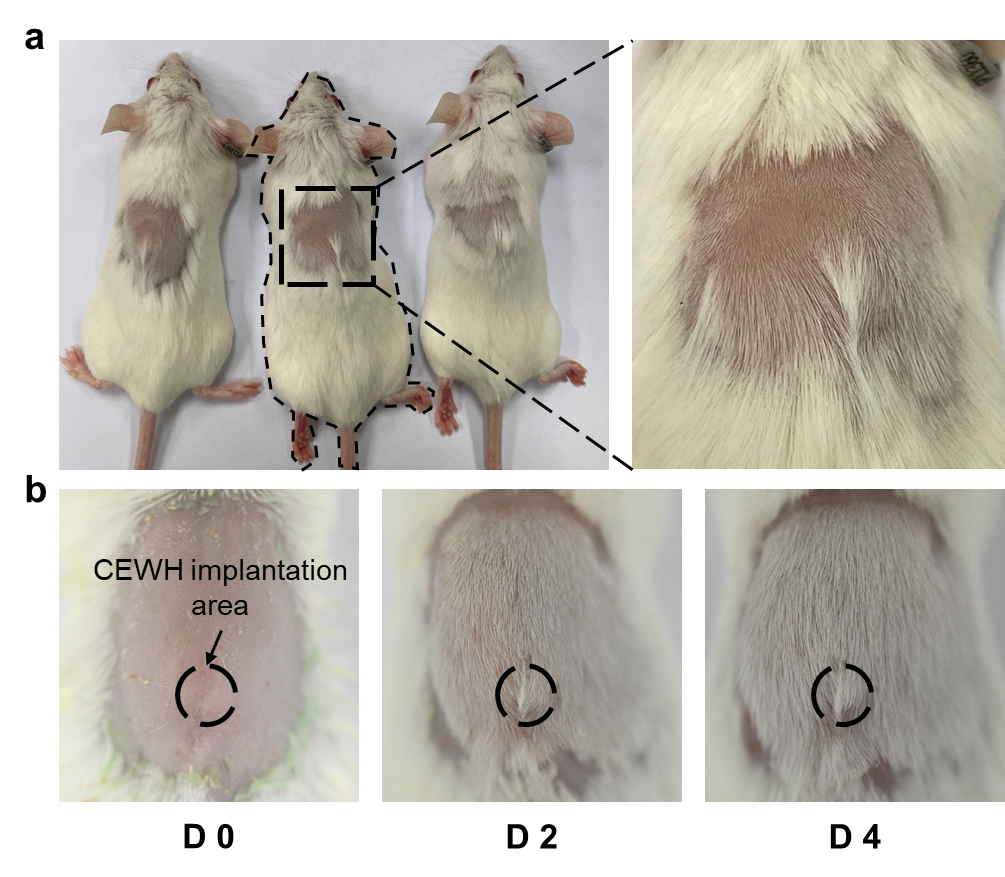


Figure S17 (a) Photographs of mice after CEWH implantation captured on day 21. The hair was removed on day 7 post implantation. (b) Photographs captured on day 21, day 23, and day 25 of mouse that underwent extensive hair removal treatment on day 21 post implantation.


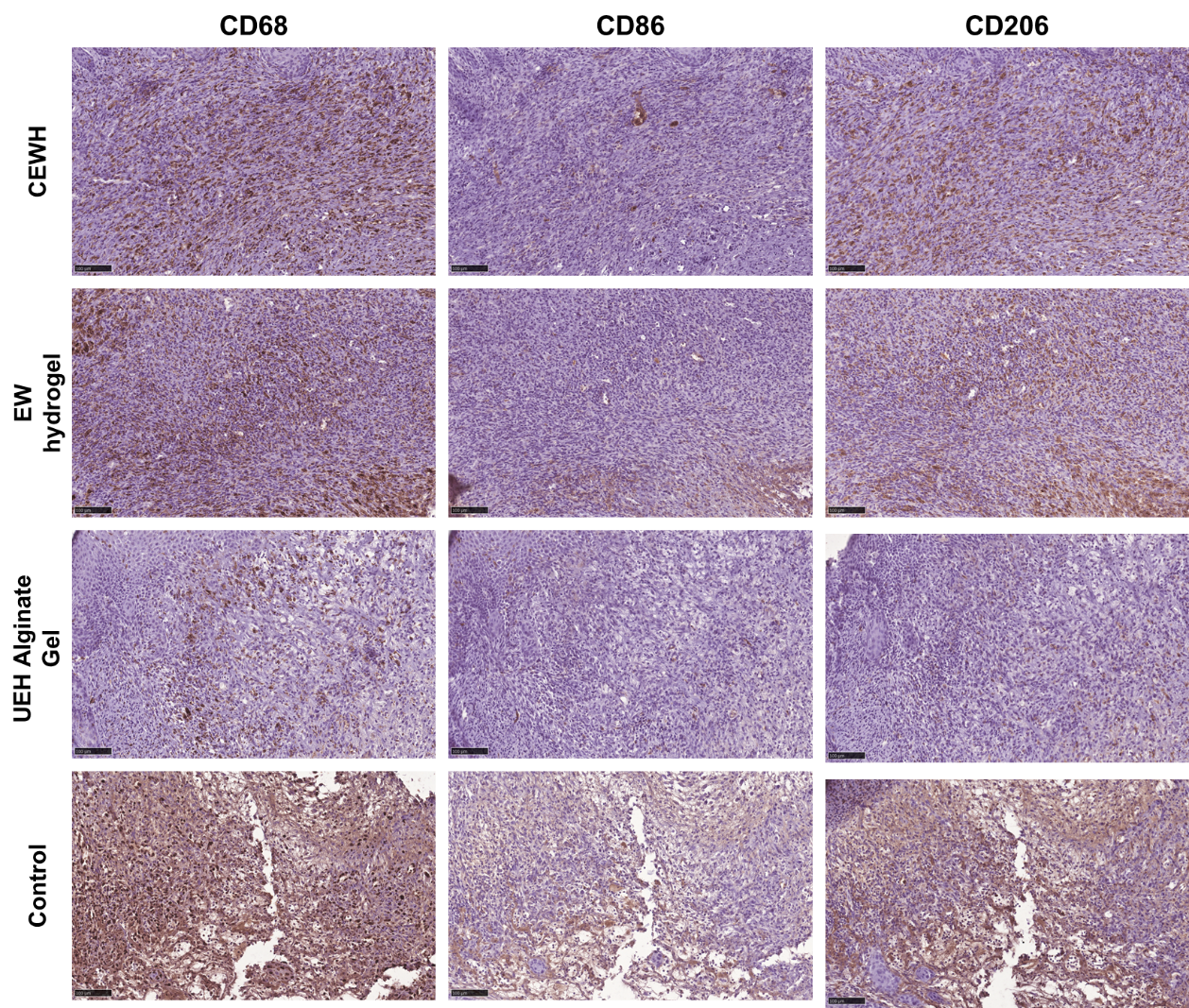


Figure S18 Immunohistochemistry (IHC) staining for four groups of CD68 (macrophage marker), CD86 (M1 macrophage marker), and CD206 (M2 macrophage marker) on day 9 post-wounding. Scale bars: 100 µm.


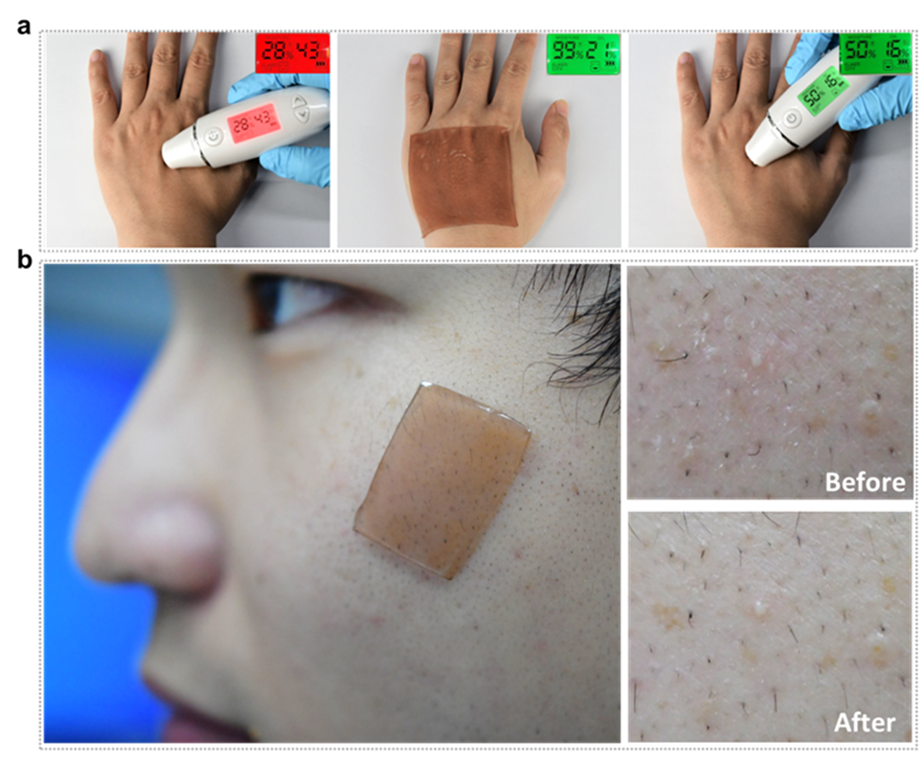


Figure S19 (a) Comparison of the oil and water contents in human hands skin before and after a 15-minute treatment with CEWH dressing. (b) A man’s facial skin before and after wearing CEWH for 15 minutes.


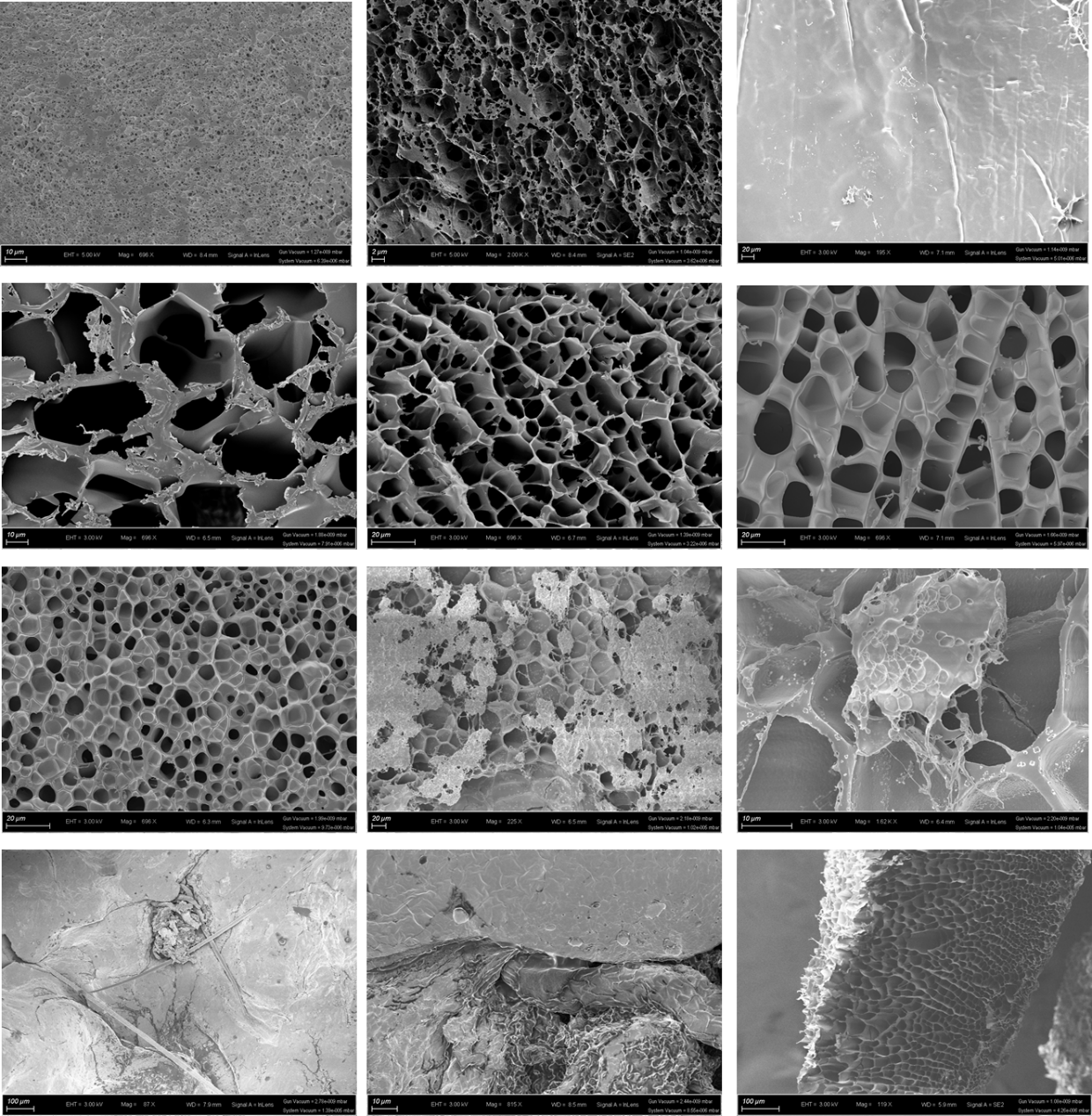


Figure S20 All original images of SEM morphology characterization.
